# Identification of actionable targets using DEPArray‐based sorting of pure carcinoma and stromal populations from formalin‐fixed paraffin‐embedded tissues followed by shallow whole‐genome sequencing

**DOI:** 10.1002/path.6469

**Published:** 2025-10-27

**Authors:** Georgios Nteliopoulos, Evie Wren, Amelia Rushton, Marc K Wadsley, Daniel Fernandez‐Garcia, Floriana Manodoro, Otis Agbaimoni, Ritika Chauhan, Zhao Cheng, Darren P Ennis, Karen Page, Rebecca C Allsopp, Joel Bautista, Ignazio Puccio, Nik Matthews, Kelly LT Gleason, Rehman Farah, Laura Kenny, Iain A McNeish, Jacqueline A Shaw, R Charles Coombes

**Affiliations:** ^1^ Department of Surgery and Cancer Imperial College London, Hammersmith Hospital Campus London UK; ^2^ Leicester Cancer Research Centre, Department of Genetics, Genomics and Cancer Sciences University of Leicester, Robert Kilpatrick Clinical Sciences Building, Leicester Royal Infirmary Leicester UK; ^3^ Genomics Facility (former TPU), Next‐generation Sequencing lab Brookes Lawley Building, Institute of Cancer Research Sutton UK; ^4^ Menarini Silicon Biosystems SpA Via Giuseppe di Vittorio, Castel Maggiore Bologna Italy; ^5^ Department of Medical Oncology Imperial College Healthcare NHS Trust, Charing Cross Hospital London UK

**Keywords:** FFPE tissue biopsy, DEPArray, breast cancer, ovarian high‐grade serous carcinoma, somatic copy number alterations (sCNA)

## Abstract

Formalin‐fixed paraffin‐embedded (FFPE) tissue specimens represent precious resources for clinical genomic profiling studies, especially when coupled with comprehensive medical records. Even though next‐generation sequencing (NGS) is an effective tool to detect somatic mutations and somatic copy number alterations (sCNA), the biggest challenges in unlocking clinically translatable genomic information from FFPE tissue are low DNA yields and degraded DNA, affected by variable formalin fixation. Another issue is that the proportion of carcinoma and other noncarcinoma cells is variable and can be confounded by intratumoral heterogeneity. To explore these challenges, we isolated pure carcinoma and stromal cells using the DEPArray™ NxT system, a microchip‐based digital sorter that allows isolation of pure, homogeneous subpopulations of cells from FFPE samples. We isolated pure carcinoma and stromal cell populations from 12 FFPE tissues, including tissues from nine primary and metastatic breast cancer and three primary ovarian high‐grade serous carcinomas. This was followed by downstream shallow whole‐genome sequencing (WGS) for copy number landscape profiling (10 samples) and/or a targeted panel for somatic mutation and sCNA analysis (seven samples), subject to cell availability. Seven out of 10 samples (even some with low tumour content or of old age) produced good‐quality genomic data, detecting sCNA in all carcinoma population samples but not in the stromal populations. Mutation analysis was performed successfully in 6/7 samples and somatic mutations were detected in all of them. Our workflow enabled the identification of clinically actionable targets, including *PIK3CA, ERBB2, FGFR1/2, CDK6, CCNE1, KRAS* amplifications and *RB, BRCA1/2* losses in patients that would direct therapy. © 2025 The Author(s). *The Journal of Pathology* published by John Wiley & Sons Ltd on behalf of The Pathological Society of Great Britain and Ireland.

## Introduction

Formalin‐fixed paraffin‐embedded (FFPE) tissue samples are essential for histological diagnosis of cancer and, since they are routinely collected, they are a valuable resource for retrospective molecular profiling studies. Next‐generation sequencing (NGS) has revolutionised cancer genomics, but the robustness of the results depends on the quality of the input DNA. Although the fixation process is essential to protect cellular morphology and protein expression, it results in the chemical modification/degradation of nucleic acids, by generating N‐methylene cross‐links in DNA, causing hydrolysis of phosphodiester bonds and reducing the stability of double‐stranded DNA [1]. Several studies have compared different methods for DNA extraction [2], sequencing library production kits [3], methods for preserving biospecimens, and other analytical parameters [4]. These methods show considerable discrepancies, implying the need for validation to ensure high‐confidence results from FFPE specimens.

FFPE samples are frequently suboptimal for obtaining good‐quality genomic data, including somatic copy number alterations (sCNA), loss of heterozygosity (LOH), and homozygous/heterozygous mutations, due to the presence of contaminating stromal cells and low percentages of carcinoma cells. As a result, the signal of genomic aberrations is diluted by noncarcinoma components in those heterogeneous samples. Microdissection can be used to enrich carcinoma cells; however, the approach is labour‐intensive, and purity is not guaranteed, especially when carcinoma cells are small or highly associated with stromal cells, such as inflammatory cells [5]. Therefore, a method that enables the isolation of epithelial carcinoma cells from the surrounding stroma is required, to improve the quality of genomic analysis. This information is particularly important when a patient relapses, since driver mutations and sCNA can evolve over time and can differ from the primary tumour. In breast cancer (BC), patients often relapse many years, or even decades after the primary diagnosis.

To address these issues, we used the DEPArray™ NxT system (Menarini Silicon Biosystems, Bryn Athen, PA, USA), which allows precise identification, characterisation, and isolation of single cells or other fluorescently‐labelled rare cells from heterogeneous samples containing up to tens of thousands of cells. The system uses image‐based selection and is based on dielectrophoresis (DEP) to isolate viable/fixed single cells and small pools [6]. The NxT instrument also includes an application for automatic selection of carcinoma and stromal populations from FFPE samples [7].

We analysed 12 small FFPE tumour biopsies, some of low tumour cellularity, that had been stored for many years. The workflow combining tissue disaggregation, digital sorting of pure carcinoma and stromal populations, followed by downstream shallow whole‐genome sequencing (WGS) and/or a targeted panel, produced robust genomic data. These data were superior or equally good to the ‘standard’ extraction methods from FFPE tissue and were able to identify actionable targets, suggesting alternative treatment options.

## Materials and methods

### Ethics approval

The study protocol was approved by the Imperial College Healthcare Tissue Bank (ICHTB) (HTA‐licence 12775; IRAS‐reference 312147, REC‐reference 22/WA/0214, project‐reference‐number: 12/WA/0196). All patients gave written informed consent prior to participation.

### Patient information

We obtained 12 FFPE tissue blocks, nine from BC patients (including primary/metastatic tumours, one pleural biopsy, one bone marrow reaming, and one lymph node biopsy) and three from ovarian high‐grade serous carcinomas (HGSC). Samples 11 and 12 were taken from the same patient.

### Sample preparation, quality control of FFPE sections, and DEPArray NxT run

We used 1–2 sections (50‐μm thick) per sample of various tumour cellularities. Tumour cellularity was estimated by a Consultant Histopathologist at Charing Cross Hospital. The disaggregation and staining of FFPE sections were conducted using the DEPArray™_FFPE_SamplePrep Kit (Menarini Silicon Biosystems [MSB], Bologna, Italy) and the integrity of cell suspensions was assessed using the DEPArray™_FFPE_QC Kit (MSB) following the manufacturer's protocol. Cells were loaded onto the DEPArray NxT cartridge (MSB), and the system's automated gating‐strategy for carcinoma/stromal cell identification was applied following the manufacturer's protocol (Supplementary materials and methods).

### Genomic analysis

The recovered cells were lysed using the DEPArray™_LysePrep Kit (MSB) and sent to the Institute of Cancer Research for library preparation and NGS. Libraries were prepared from 10 samples using the DEPArray™_LibPrep Kit (MSB) and low‐pass NGS performed for sCNA analysis. For seven samples (overlap in five patients), we prepared libraries using the DEPArray™_OncoSeek_Cancer_Hotspot_gene_panel (MSB), which is an amplicon‐based panel of 63 genes of interest, including 56 assessed for mutations and 19 for sCNA. The samples were run on an Illumina_NextSeq_2000 sequencer (Illumina, Cambridge, UK) following the manufacturer's protocol. The generated fastq files were aligned to the hg19 reference using BWA‐MEM [8], and WIG files were created from the resulting BAM files using HMMcopy readcounter (https://github.com/shahcompbio/hmmcopy_utils, 30 July 2025) with bin sizes of 0.5 MB and a stringent mapping quality filter of 20. sCNA analysis was performed using IchorCNA v0.2.0 (https://github.com/broadinstitute/ichorCNA, 30 July 2025) with default settings and no baseline to predict sample tumour fraction (TFx) and CN counts [9]. Quality control (QC) metrics required sequencing data to exceed > 100,000 productive reads with a median‐absolute‐pairwise difference (MAPD) < 0.3, which provides a measure of read coverage noise. Overlap of sCNA calls with 93 BC‐related genes [10] was performed using bedtools (v2.31.0) [11]. Analysis of the OncoSeek panel fastq files was performed using the cloud‐based MSBiosuite (MSB). For the workflow of FFPE tissue coring, tumour DNA was extracted using the GeneRead™_DNA_FFPE kit (Qiagen, Hilden, Germany), while Ion S5 XL sequencing platform (Thermo Fisher Scientific, Altrincham, UK) was used for sWGS. The sequencing data (fastq files) will be publicly available via the European Genome‐phenome Archive at the European Bioinformatics Institute (https://ega-archive.org).

## Results

### Recovery of FFPE subpopulations by DEPArray

**Figure 1:**
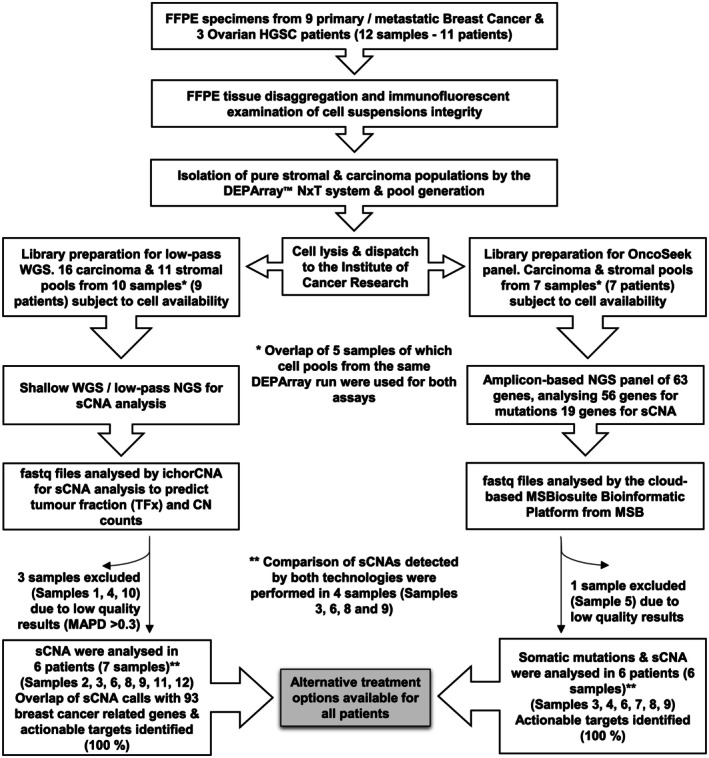
REMARK diagram summarising the workflow, the samples used for each analysis, and the samples excluded. Patient samples were obtained from Imperial College Healthcare Tissue Bank. The DEPArray™ FFPE SamplePrep Kit was used for FFPE tissue disaggregation and immunofluorescent staining was and the DEPArray™ FFPE QC Kit for the examination of cell suspensions integrity. The number of cells pooled per DEPArray run was calculated by the formula: number of cells = EAT/(QC score x ploidy), where EAT is the effective amplifiable template minimum for each downstream application, QC score the DEPArray‐QC score and ploidy the DNA Index x 2. The DEPArray™ LysePrep Kit was used for cell lysis. The DEPArray™ LibPrep Kit and the DEPArray™ OncoSeek Cancer Hotspot gene panel were used for library preparation for the low‐pass shallow whole‐genome sequencing (sWGS) and the OncoSeek panel, respectively. Both sWGS and targeted OncoSeek panel were run by Illumina NextSeq 2000. QC metrics for sWGS required > 100,000 productive reads and MAPD < 0.3. WGS. MSBiosuite platform was from Menarini Silicon Biosystems (MSB).

Details of carcinoma and stromal fractions isolated from different FFPE tissues are summarised in the REMARK diagram in Figure 1 and supplementary material, Table S1. The paraffin‐embedded tissues were stored for a median of 7 years (range 3–10) prior to analysis. Tumour content was estimated at between 10% and 85% by a Consultant Histopathologist. QC scores were assessed by the DEPArray™_FFPE_QC Kit (DEPArray‐QC) and ranged between 0.06–1.07 (median 0.36). No significant correlation was observed between sample age and DEPArray‐QC score (*r* = 0.37, *p* = 0.3). Stromal and carcinoma populations were recovered from each sample using the automated selection process (supplementary material, Figure S1). When DEPArray generated more than one subgroup of carcinoma cells based on the DNA index (DI), these were pooled separately. Based on the DEPArray‐QC scores, the ploidy (DI × 2), and the effective amplifiable template (EAT) minimum for each downstream application (supplementary material, Table S2), the calculated number of cells/pool was displayed next to the actual number of cells/pool (supplementary material, Table S1). In 10/42 (24%) of pools/libraries, significantly fewer cells were pooled than required; however, these were still included in the analysis.

### Low‐pass (shallow) whole‐genome sequencing (WGS)

**Figure 2:**
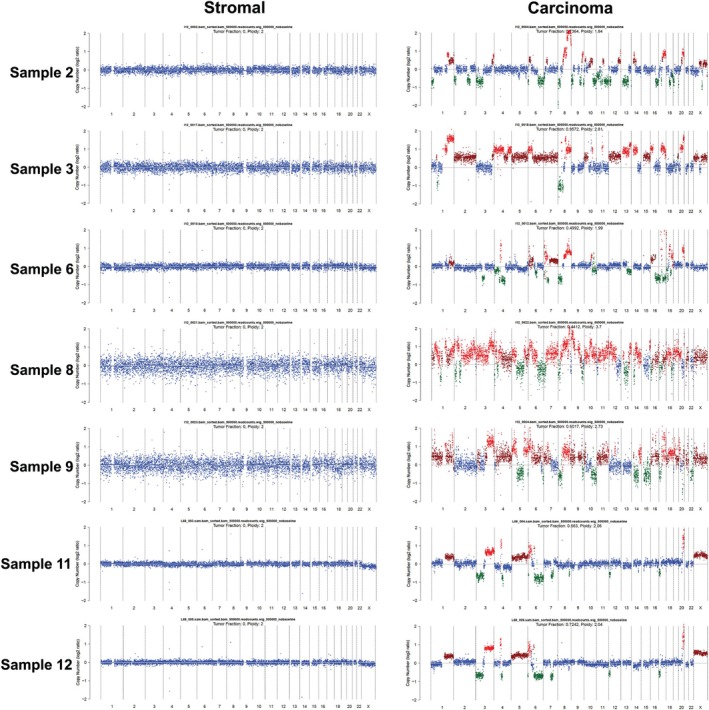
Representative copy number (CN) profiles of stromal and carcinoma populations from all samples that passed next‐generation sequencing (NGS) quality metrics with MAPD < 0.3. Tumour fraction (TFx) and ploidy, calculated by ichorCNA, are shown in each copy number (CN) profile, using the 0.5 MB tile size parameter. Losses (CN = 1) are highlighted with green, gains (CN = 3) with dark red, amplifications (CN ≥4) with red and the baseline (CN = 2) with blue.

Libraries from 10/12 samples consisting of 16 carcinoma and 11 stromal pools were prepared for sCNA analysis (due to cell number limitations, two of the samples were used only for the OncoSeek panel). All libraries apart from samples 1 and 10 had median‐absolute‐pairwise difference (MAPD) < 0.3, indicating good‐quality data (Table 1, Figure 2 and supplementary material, Figure S2), showing representative copy number profiles of samples with MAPD < 0.3 and MAPD > 0.3, respectively. Overall, there was a moderate negative correlation between DEPArray‐QC score and GC‐corrected MAPD score (*r* = −0.47, *p* = 0.01). The two samples with high MAPD scores had DEPArray‐QC < 0.2. The median number of cells/library was 51 (range 12–413), greater compared to that needed according to EAT (median, 42; range 5–625). There was a strong positive correlation between the number of cells and MAPD score (*r* = 0.74, *p* < 0.0001), demonstrating that high numbers of cells/library imply low DEPArray‐QC score, and therefore poor‐quality NGS. The third sample with MAPD > 0.3 was sample four (supplementary material, Figure S2), with libraries of low cell number. Although the number of cells used were 17 and 20 for stromal and carcinoma, respectively, which were close to the required number i.e. 20, the high DEPArray‐QC score of 0.74 may have led to an underestimate of the number of cells needed. Seven other libraries with small numbers of cells (12–30 cells) had MAPD < 0.3, and there was no significant difference between the failure rate of the libraries of ≤ 30 cells (2/9) and those > 30 cells (5/18) (Fisher‐exact test; *p* = 1). Regarding the effect of FFPE sample storage time, there was no correlation between storage time and MAPD (*r* = −0.14, *p* = 0.5), or the DEPArray‐QC score (*r* = 0.07, *p* = 0.7). For three samples, which had been stored for up to 5 years, 2/4 (50%) pools/libraries gave an MAPD > 0.3. For ten older samples (storage age ranging 6–10 years), the success rate in library creation was 72% (13/18), but this did not reach statistical significance (Fisher‐exact test; *p* = 0.27).

**Table 1:** Summary of the sCNA analysis

Patient sample	Sample ID	GC map correction MAPD	DEPArray FFPE QC kit score	No. cells/ library	Cell type	Tumour Fraxion (TFx)	Ploidy	Autosome CN count	Stratton (2016) Genes gain and loss
s2	I12_0001	0.101	0.56	31	stromal	0	1.997	0	
	I12_0002	0.110	0.56	68	stromal	0	1.997	0	
	I12_0003	0.167	0.56	22	carcinoma	0.615	1.897	38	*ASXL1, BRCA1, CCND1, ERBB2, FOXA1, GNAS, MYC, PIK3CA, PREX2, PTEN, TBX3, ZNF217* *AFDN, ARID1B, ATM, BRCA2, CBFB, CDH1, CDKN1B, CTCF, ECT2L, ESR1, FGFR2, FOXP1, HRAS, KRAS, MED23, MEN1, MLH1, NOTCH1, PBRM1, PMS2, RB1, RHOA, SETD2, SMAD4, SMARCA4, SPEN, STK11*
	I12_0004	0.116	0.56	65	carcinoma	0.736	1.838	51	*ASXL1, ATRX, BRCA1, CCND1, ERBB2, FOXA1, GNAS, MYC, PHF6, PIK3CA, PREX2, PTEN, STAG2, TBX3, ZNF217 AFDN, ARID1B, ATM, BRCA2, CBFB, CDH1, CDKN1B, CTCF, ECT2L, ESR1, FGFR2, FOXP1, HRAS, KRAS, MED23, MEN1, MLH1, NF1, NOTCH1, PBRM1, PMS2, RB1, RHOA, SETD2, SMAD4, SMARCA4, SPEN, STK11*
	I12_0005	0.155	0.56	16	carcinoma	0.419	2.026	26	*ASXL1, BRCA1, CCND1, ERBB2, FOXA1, GNAS, MYC, PIK3CA, PREX2, PTEN, TBX3, ZNF217* *ATM, BCOR, BRCA2, CBFB, CDH1, CTCF, FGFR2, FOXP1, MLH1, NOTCH1, PBRM1, RB1, RHOA, SETD2, SMARCA4, SPEN, STK11, USP9X*
	I12_0006	0.102	0.56	39	carcinoma	0.465	2.078	47	*ASXL1, BRCA1, BUB1B, CCND1, CCND3, CREBBP, ERBB2, ERBB3, ERCC4, FBXW7, FOXA1, GATA3, GNAS, IGF1R, KMT2D, MDM2, MYC, PALB2, PDGFRA, PIK3CA, PREX2, PTEN, TBX3, TET2, ZNF217* *ATM, BCOR, BRCA2, CBFB, CDH1, CDKN2A, CDKN2B, CTCF, FGFR2, FOXP1, HRAS, KDM6A, MEN1, MLH1, NOTCH1, PBRM1, RB1, RHOA, RUNX1, SETD2, SMAD4, SMARCA4, SPEN, STK11, USP9X*
s3	I12_0017	0.161	0.28	51	stromal	0	1.999	0	
	I12_0018	0.121	0.28	51	carcinoma	0.957	2.812	34	*AFDN, APC, ARID1B, ASXL1, BCOR, BRAF, BRCA1, BRCA2, BUB1B, CASP8, CCND1, CCND3, CDK6, CDKN1B, CNOT3, CREBBP, CUX1, DNMT3A, ECT2L, EGFR, ERBB2, ERBB3, ERCC4, ESR1, FBXW7, FGFR1, FOXA1, GATA3, GNAS, IGF1R, KDM6A, KMT2C, KMT2D, KRAS, MAP3K1, MDM2, MED23, MEN1, MSH2, MYC, NF1, NOTCH2, PALB2, PDGFRA, PHF6, PIK3CA, PIK3R1, PMS2, PRDM1, PTEN, RB1, SF3B1, STAG2, TBX3, TET2, USP9X, ZNF217*
s6	I12_0010	0.094	0.35	231	stromal	0	1.995	0	
	I12_0011	0.125	0.35	78	carcinoma	0.675	1.987	21	*BRAF, CDK6, CREBBP, CUX1, ERBB2, ERCC4, GNAS, KMT2C, MYC, NF1, PALB2, PMS2, PREX2, ZNF217* *BRCA1, CBFB, CDH1, CTCF, EGFR, FGFR1, FOXP1, MAP2K4, NCOR1, PDGFRA, TET2, TP53*
	I12_0012	0.084	0.35	117	carcinoma	0.499	1.986	30	*BRAF, CCND3, CDK6, CREBBP, CUX1, ERBB2, ERCC4, GNAS, KMT2C, MYC, NF1, PALB2, PREX2, ZNF217* *BRCA1, CBFB, CDH1, CTCF, EGFR, FGFR1, FGFR2, FOXP1, MAP2K4, NCOR1, PDGFRA, PMS2, PTEN, SMAD4, TET2, TP53*
	I12_0013	0.080	0.35	12	carcinoma	0.103	1.986	20	*AKT2, BRAF, CCNE1, CDK6, CIC, CNOT3, CUX1, ERBB2, ERBB3, GNAS, KMT2C, KMT2D, KRAS, MDM2, MYC, NF1, PMS2, PREX2, RUNX1, SMARCA4, STK11, ZNF217* *APC, BRCA1, CBFB, CDH1, CTCF, FGFR1, FOXP1, MAP2K4, NCOR1, PDGFRA, PIK3R1, TET2, TP53*
s8	I12_0021	0.279	0.62	35	stromal	0	1.996	0	
	I12_0022	0.246	0.62	25	carcinoma	0.441	3.7	68	*AKT1, AKT2, ARID1A, ASXL1, ATM, ATR, ATRX, BCOR, BRAF, BRCA1, CASP8, CBFB, CBLB, CCND1, CCND3, CCNE1, CDH1, CDK6, CDKN1B, CDKN2A, CDKN2B, CIC, CNOT3, CTCF, CUX1, EGFR, ERBB3, FBXW7, FGFR1, FGFR2, FOXA1, FOXP1, GATA3, IGF1R, KDM6A, KMT2C, KMT2D, KRAS, MDM2, MEN1, MLH1, MSH2, MYC, NOTCH2, NRAS, PALB2, PBRM1, PDGFRA, PHF6, PIK3CA, PREX2, PTEN, RHOA, RUNX1, SETD2, SF3B1, SMAD4, SMARCA4, SPEN, STAG2, TET2, USP9X, ZFP36L1* *AFDN, APC, ARID1B, BRCA2, CREBBP, DNMT3A, ECT2L, ERBB2, ERCC4, ESR1, MAP2K4, MAP3K1, MED23, NCOR1, NF1, PIK3R1, PMS2, PRDM1, RB1, TP53*
s9	I12_0023	0.266	1	24	stromal	0	2.002	0	
	I12_0024	0.183	1	25	carcinoma	0.602	2.732	49	*AKT1, AKT2, ARID1A, ARID1B, ATM, ATR, ATRX, BCOR, BRCA1, CBFB, CBLB, CCND1, CCND3, CCNE1, CDH1, CDKN2A, CDKN2B, CIC, CNOT3, CREBBP, CTCF, ECT2L, EGFR, ERBB2, ERCC4, ESR1, FBXW7, GATA3, GNAS, HRAS, KDM6A, MDM2, MED23, MEN1, MLH1, MYC, NOTCH1, NOTCH2, NRAS, PALB2, PDGFRA, PHF6, PIK3CA, PMS2, PRDM1, PREX2, RUNX1, SMAD4, SMARCA4, SPEN, STAG2, TET2, USP9X, ZNF217* *AFDN, APC, BUB1B, FGFR1, FGFR2, FOXA1, IGF1R, MAP2K4, MAP3K1, NCOR1, NF1, NF2, PIK3R1, PTEN, TP53, XBP1, ZFP36L1*
s11	L68_003	0.05081	0.36	53	stromal	0	1.998	0	
	L68_004	0.06768	0.36	46	carcinoma	0.663	2.062	15	*APC, ATR, ATRX, BCOR, CBLB, GNAS, KDM6A, MAP3K1, PHF6, PIK3CA, PIK3R1, STAG2, USP9X, ZNF217* *AFDN, ARID1B, CDK6, CDKN1B, CUX1, ECT2L, ESR1, FOXP1, MAP2K4, MED23, MLH1, NCOR1, PBRM1, PRDM1, RHOA, SETD2, TP53*
s12	L68_005	0.04726	0.31	60	stromal	0	1.999	0	
	L68_006	0.06017	0.31	47	carcinoma	0.7242	2.039	14	*APC, ATR, ATRX, BCOR, CBLB, GNAS, KDM6A, MAP3K1, PHF6, PIK3CA, PIK3R1, STAG2, USP9X, ZNF217* *AFDN, ARID1B, CDK6, CDKN1B, CUX1, ECT2L, ESR1, FOXP1, MAP2K4, MED23, MLH1, NCOR1, PBRM1, PRDM1, RHOA, SETD2, TP53*

*Note*: GC‐corrected MAPD score, DEPArray‐FFPE QC score, the number of cells/library, the cell type, the tumour fraction (TFx) and ploidy as calculated by ichorCNA, the number of sCNA called and the overlap of sCNA with 93 breast cancer related genes (Stratton, 2016) [10] are shown. Genes with gains/amplifications are highlighted red, genes with losses are highlighted green.

Table 1 summarises the data from the sCNA analysis. All eight stromal samples had no sCNA, whereas sCNA were detected in all 12 carcinoma samples, demonstrating a good separation of the two subpopulations. The mean number of sCNA in the samples with MAPD > 0.3 was 36 (range 14–68).

**Figure 3:**
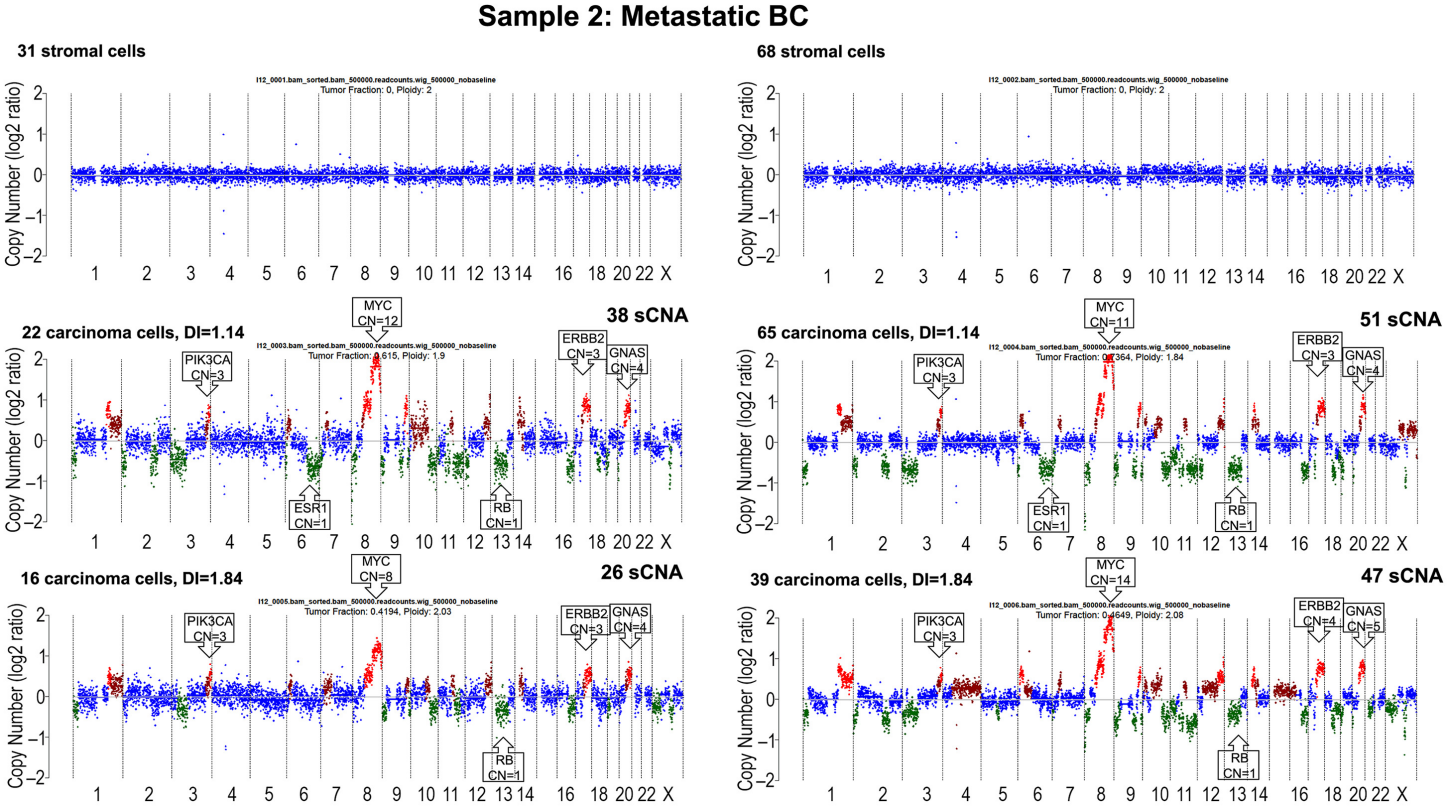
Copy number (CN) profiles of stromal and carcinoma populations (with two different DNA Indexes (DI)) using a lower (left panel) or higher (right panel) number of cells per library. Tumour fraction (TFx) and ploidy, calculated by ichorCNA, the number of cells, the DNA Index (DI), the number of somatic copy number alterations (sCNA) called, the breast cancer‐related genes overlapping with sCNA and the copy number (CN) for those genes versus the baseline (CN = 2) are shown in each CN profile. Losses (CN = 1) are highlighted with green, gains (CN = 3) with dark red, amplifications (CN ≥4) with red, and the baseline (CN = 2) with blue.

Sample 2 was resected from an ER+ metastatic BC (mBC) patient, whilst on endocrine therapy. This 9‐year‐old tissue had a tumour cellularity of 75%, and a DEPArray‐QC score of 0.56. We analysed six libraries in total and compared two libraries from each subgroup (stromal, carcinoma DI = 1.14 and carcinoma DI = 1.84) based on the lower and higher cell number/library (Figure 3). Libraries with more carcinoma cells generated better‐quality data, as shown by the MAPD scores (Table 1), the increased TFx, and a greater number of called sCNA (Figure 3). The most amplified regions included *MYC*, *GNAS*, whereas gains in *PIK3CA*, *ERBB2* and losses in *ESR1, RB1*, *BRCA2* were also observed. Aneuploid carcinoma cells of DI = 1.14 and DI = 1.84 showed similar sCNA calls of 51 and 47, respectively. However, the library with DI = 1.84 had fewer cells as DNA input, affecting the signal amplitudes. Interestingly, ichorCNA performed ploidy fitting and found that the optimal fitting for DI = 1.84 (and for DI = 1.14) was pseudo‐diploid, rather than aneuploid, as guided by the DEPArray‐DI values (supplementary material, Figure S3). The sCNA profile comparison between the two populations with different DI shows many similarities, but also some differences.

We also analysed a metastatic pleural biopsy containing ER+ mBC (Sample 3) collected 5 years after the diagnosis of primary BC (pBC), while the patient was on endocrine therapy. The sample had a tumour cellularity of 85% and DEPArray‐QC score of 0.28 (supplementary material, Figure S4). The carcinoma population had a very high TFx (0.96), with 34 sCNA and regions containing *FGFR1, CCND1, GNAS*, and *BRAF* the most amplified. *ERBB2* amplification may have informed treatment choices. In this case, ichorCNA fitted the carcinoma cells as aneuploid in the range of triploid, which agrees with the one suggested by DEPArray‐DI (1.41).

We also analysed pBC samples. Sample 6, a 7‐year‐old ER + HER2+ tissue had a tumour cellularity of 80% and DEPArray‐QC score of 0.35 (supplementary material, Figure S5). All three carcinoma populations of different DI showed similar profiles with sCNA, including *MYC, GNAS, BRAF* amplifications and, as expected, *ERBB2* high‐level amplification. Other sCNA include *CDK6* gain as well as *BRCA1* loss. Once again, ichorCNA fitted the three subpopulations as pseudo‐diploid despite their differing DEPArray‐DI scores (DIs = 0.5, 1.05, 1.8). The first two subpopulations had very similar sCNA profiles, while the third had many differences; however, due to the low cell number the signal was weak.

**Figure 4:**
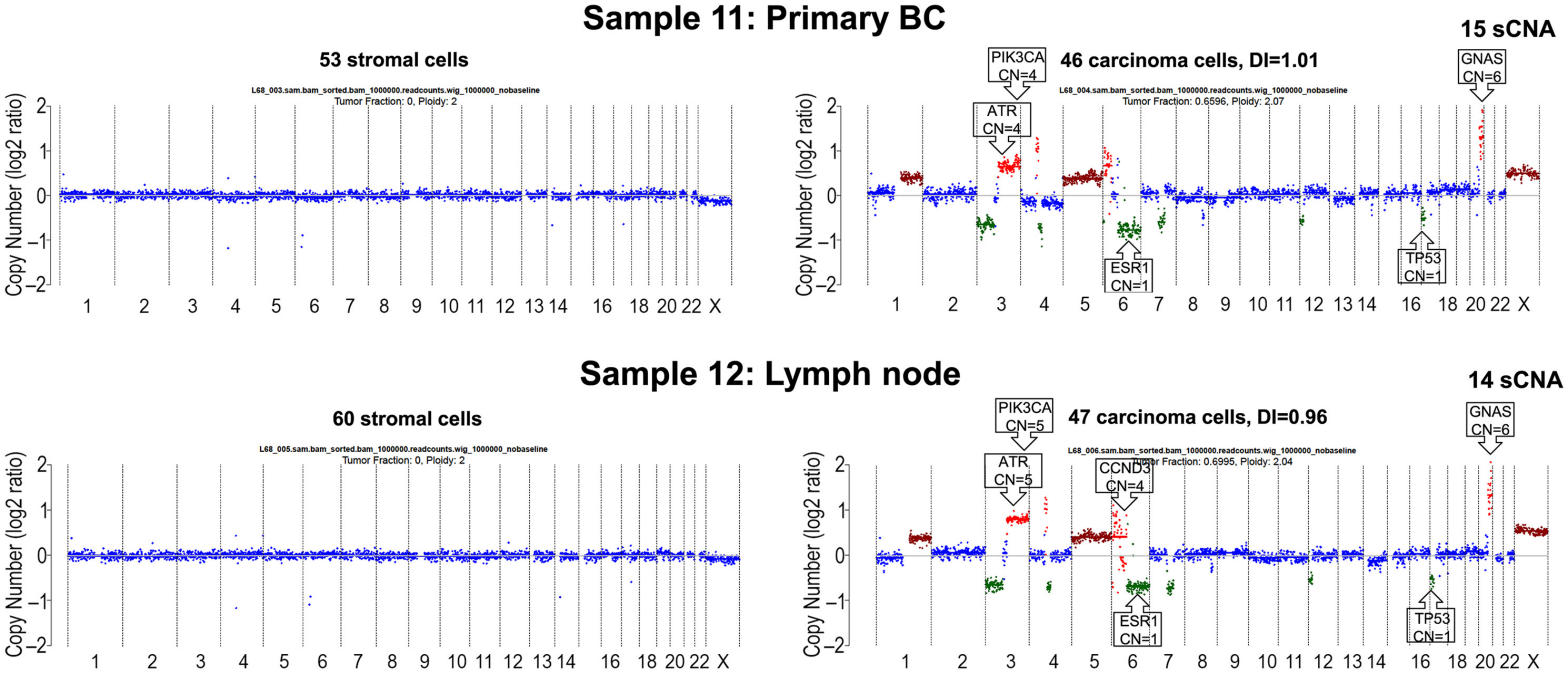
Copy number profiles of stromal and tumour populations from different tissues from the same patient (primary breast and lymph node from the same timepoint). Tumour fraction (TFx) and ploidy, calculated by ichorCNA, the number of cells, the DNA Index (DI), the number of somatic copy number alterations (sCNA) called, the breast cancer‐related genes overlapping with sCNA, and the copy number (CN) for those genes versus the baseline (CN = 2) are shown in each CN profile. Losses (CN = 1) are highlighted with green, gains (CN = 3) with dark red, amplifications (CN ≥4) with red, and the baseline (CN = 2) with blue.

Sample 11 was from a primary ER + HER2‐ breast tumour. Previous samples described had tumour cellularities > 75%, whereas this sample had a low tumour cellularity (10%). We also analysed, from the same patient, a positive for cancer lymph node (Sample 12), again of low tumour cellularity (15%), sampled at the time of breast resection. Samples 11 and 12 were 5 years old, with DEPArray‐QC scores of 0.36 and 0.31, respectively. These samples were pseudo‐diploid and showed very similar profiles, with a similar number of sCNA calls, including regions containing *ATR, GNAS*, *PIK3CA* amplifications and *ESR1*, *TP53* losses (Figure 4).

To assess the benefits of the DEPArray workflow in a low‐tumour‐cellularity sample, we compared the same samples with the routine workflow of FFPE tissue cores taken from representative tumour areas, a technique used to enrich for carcinoma populations. An area of tumour tissue was identified in the FFPE block by a Consultant Histopathologist and sampled using a 1‐mm tissue microarray (TMA) needle core. Tumour DNA was extracted using the GeneRead™_DNA_FFPE kit; Ion Torrent sWGS was conducted and ichorCNA was used for sCNA analysis. Interestingly, when we ran the same parameters (1‐MB tiles), we found that the DEPArray workflow provided lower MAPD scores, higher TFx, and more sCNA, especially in breast tissue, providing robust genomic data in tissues with very low tumour cellularity (Tables 2 and 3). The most striking difference involved the number of gene losses.

**Table 2:** sCNA analysis following the standard coring workflow (University of Leicester). Tumour tissue was identified in the formalin‐fixed, paraffin‐embedded (FFPE) block by a consultant histopathologist and sampled using a 1 mm TMA needle core. Tumour DNA was extracted using the GeneRead™ DNA FFPE kit, followed by Ion Torrent S5 next‐generation sequencing (NGS)

Sample ID	GC‐Map correction MAPD	Tumour Fraction (TFx)	Autosome CN count	Stratton (2016) Genes Gain	Stratton (2016) Genes Loss
S11 (breast tissue)	0.186	0.36	3	*ASXL1, ATR, ATRX, BCOR, CBLB, GNAS, KDM6A, PHF6, PIK3CA, STAG2, USP9X, ZNF217*	
S12 (lymph node)	0.161	0.53	7	*APC, ATR, ATRX, BCOR, CBLB, CCND3, GNAS, KDM6A, MAP3K1, PHF6, PIK3CA, PIK3R1, STAG2, USP9X, ZNF217*	*ARID1B, ECT2L, ESR1, FOXP1, MED23, MLH1, PBRM1, PRDM1, RHOA, SETD2*

*Note*: Somatic copy number alterations (sCNA) analysis was performed by ichorCNA (1 MB bin size) to calculate GC‐corrected MAPD scores and predict tumour fraction (TFx) and copy number (CN) counts. The overlap of sCNA with 93 breast cancer related genes (Stratton, 2016) [10] is shown where identified genes with gains/amplifications without overlap with those from Table 3 (DEPArray workflow) are highlighted in red.

**Table 3:** sCNA analysis following the DEPArray workflow (Imperial College/Institute of Cancer Research). Formalin‐fixed, paraffin‐embedded (FFPE) block sections were dissociated and cell suspensions immunofluorescently stained by DEPArray™ SamplePrep. Cells were ran on a DEPArray™ NxT system followed by DEPArray™ LibPrep and LowPass‐whole‐genome sequencing (WGS) using Illumina NextGene 2000 NGS.

Sample ID	GC‐Map correction MAPD	Tumour Fraction (TFx)	Autosome CN count	Stratton (2016) Genes Gain	Stratton (2016) Genes Loss
S11 (breast tissue)	0.068	0.66	13	*APC, ATR, ATRX, BCOR, CBLB, GNAS, KDM6A, MAP3K1, PHF6, PIK3CA, PIK3R1, STAG2, USP9X, ZNF217*	*AFDN, ARID1B, CDK6, CDKN1B, CUX1, ECT2L, ESR1, FOXP1, MAP2K4, MED2, MLH1, NCOR1, PBRM1, PRDM1, RHOA, SETD2, TP53*
S12 (lymph node)	0.060	0.70	12	*APC, ATR, ATRX, BCOR, CBLB, CCND3, GNAS, KDM6A, MAP3K1, PHF6, PIK3CA, PIK3R1, STAG2, USP9X, ZNF217*	*AFDN, ARID1B, CDK6, CDKN1B, CUX1, ECT2L, ESR1, FOXP1, MAP2K4, MED2, MLH1, NCOR1, PBRM1, PRDM1, RHOA, SETD2, TP53*

*Note*: Somatic copy number alterations (sCNA) analysis was performed by ichorCNA (1 MB bin size) to calculate GC‐corrected MAPD scores and predict tumour fraction (TFx) and copy number (CN) counts. The overlap of sCNA with 93 breast cancer related genes (Stratton, 2016) [10] is shown where genes with gains/amplifications and losses without overlap with those from Table 2 (Standard coring workflow) are highlighted in red and green, respectively.

Next, we investigated the potential use of this workflow in other tissues by examining three ovarian HGSC, two of which had MAPD < 0.3. Sample 8, a 3‐year‐old tissue, had a tumour cellularity of 80% and DEPArray‐QC score of 0.64, whereas 10‐year‐old sample 9 had a tumour cellularity of 70% and DEPArray‐QC score of 1.07. Based on the DEPArray‐QC scores, 25 carcinoma population cells were pooled from both samples. As demonstrated in the supplementary material, Figure S6, carcinoma populations of both samples had ~50 sCNA, with *PIK3CA, MYC* amplifications and *TP53* loss present. In addition, amplifications were identified in *CCNE1*, *KRAS* and loss of *BRCA2* in sample 8, and *ERBB2* amplification and *CCNE1* gain in sample 9. As we used < 30 cells, the results were less robust, since the MAPD scores for both samples were between 0.18 and 0.28. Both samples were aneuploid by their DI (1.51 for sample 8; 1.64 for sample 9) and were also calculated aneuploid by ichorCNA in the range of triploid cells. We compared our data with data derived from the same tissue samples after microdissection using both the QDNAseq package (bin size of 30 kB) and ichorCNA. The sCNA profiles were found to be comparable (TFx = 0.51 and TFx = 0.59 for microdissection versus TFx = 0.44 and TFx = 0.60 for DEPArray for samples 8 and 9, respectively) (supplementary material, Figure S7).

The actionable targets identified by the sCNA analysis are summarised in Table 4, together with alternative sCNA‐guided treatment.

**Table 4:** Summary of the patients' disease, treatment at the time of sampling, the somatic copy number alterations (sCNA) that affected the genes, and the treatment that may have been given to patients had the sCNA results been known at the time.

Patient	Disease	Tx at time of sample	sCNA	sCNA guided Tx
s2	ER+PR+HER2− mBC	Endocrine therapy	*ERBB2, PIK3CA, RB1, ESR1, BRCA2*	HER2 targeted therapy, PIK3CA inhibitors, CDK4/6 inhibitors resistance, potential endocrine resistance, PARP inhibitors
s3	ER+PR+HER2− mBC	Everolimus and Exemestane	*FGFR1, ERBB2*	FGFR inhibitors, HER2 targeted therapy
s6	ER+PR+HER2+ pBC	Neoadjuvant HER2 targeted therapy	*ERBB2, CDK6, BRCA1*	Potential future CDK4/6 inhibitors resistance, PARP inhibitors
s8	pHGSC	Off treatment	*CCNE1, KRAS, BRCA1, PIK3CA*	Platinum chemotherapy resistance, PARP inhibitors, PIK3CA inhibitors
s9	pHGSC	Off treatment	*ERBB2, PIK3CA*	HER2 targeted therapy, PIK3CA inhibitors
s11/s12	ER+PR+HER2− pBC	Off treatment	*PIK3CA, ESR1*	Potential future PIK3CA inhibitors, potential endocrine resistance

*Note*: Gains/amplifications are highlighted with red and deletions are highlighted with green.

### Comparison of the sCNA calling of the shallow WGS with the DEPArray OncoSeek panel

In seven samples (samples 3–9) we used isolated stromal and carcinoma populations from the same DEPArray‐run to perform genomic analysis using the DEPArray OncoSeek panel, which allows simultaneous analysis of variants and sCNA in cancer‐associated genes. In five samples we performed both analyses, but samples 4 and 5 did not pass QC metrics for low‐pass and OncoSeek, respectively. Therefore, overall, we compared four samples (samples 3, 6, 8, 9). The panel allowed sCNA analysis of 19 genes, of which 11 were also present in the 93 BC‐related protein‐coding genes with probable driver mutations [10]: *BRAF, CCND1, CDK6, EGFR, ERBB2, FGFR1, FGFR2, KRAS, MYC, PDGFRA*, and *PIK3CA*. All seven amplifications identified by the OncoSeek panel were also detected by the low‐pass sequencing, including *FGFR1, CCND1* in sample 3, *BRAF, ERBB2* in sample 6, *MYC* and *ALK* (not present in the 93 genes but located in the chromosome 2 region with high‐level amplification) in sample 8, and *ERBB2* in sample 9 (supplementary material, Figure S8). Using less stringent criteria including all the gains, amplifications, and losses by low‐pass and sCNA fold changes > 1.5 for gains and < 0.5 for losses between stromal and carcinoma populations for OncoSeek (supplementary material, Table S3), we found substantial agreement (82% agreement, Cohen's *k* = 0.62) in samples 6, 9, and slight agreement (36% agreement, *k* = 0.07; 27% agreement; *k* = 0.04) in samples 3 and 8, respectively.

In addition, the mutation analysis performed in six samples (excluding sample 5) for 56 genes included in the panel demonstrated somatic variants with variant allele frequency (VAF) ranging from 5% to 100% (supplementary material, Table S4). All samples had at least one somatic variant (median 3.5, range 1–6), found in 15 genes, with *TP53* being the most frequently mutated (5/6 patients), followed by *MAP2K1* and *FLT3* (2/6 patients).

## Discussion

Tissue biopsies are the gold‐standard material for histopathologists to establish a cancer diagnosis, and FFPE tissue remains essential for performing molecular profiling. However, ~30% of cancer biopsies yield samples that fail quality controls (i.e. insufficient material, low tumour cellularity, poor DNA quality) [12, 13]. Here, we describe a method that utilised FFPE tissue disaggregation and fluorescent staining combined with DEPArray™ sorting, followed by downstream genomic analysis, in which historical FFPE tissues of low tumour cell count and cellularity could be sorted into pure cell populations, generating results that may aid clinicians in selecting therapies for pBC/mBC and HGSC patients.

Flow cytometry‐based sorting of stromal and carcinoma cells from FFPE samples dates back to the original work of Corver *et al* [14]. DEPArray has been utilised for isolation of various pure populations or single cells from FFPE and fresh/frozen tissues coupled with a genomic downstream application, including Hodgkin and Reed–Sternberg cells [15], B‐cells [16], glioblastoma cells of different origins [17], uveal melanoma cells, and infiltrating immune cells [18]. Moreover, pure carcinoma cells have been sorted from FFPE oesophageal and lung adenocarcinoma tissues, detecting hyperdiploid/pseudo‐diploid subclones with different mutational loads, and/or additional sCNA and more somatic mutations (with higher VAF), respectively, identified from DEPArray‐sorted compared to unsorted cells [19, 20]. Some double Cytokeratin+/Vimentin+ cells were also found [20], similar to colorectal cancer [21], suggesting the presence of epithelial‐to‐mesenchymal transition (EMT) subpopulations.

In the most extensive study utilising DEPArray, Bolognesi *et al* included FFPE sections from ovarian and pancreatic cancers, and FFPE cores from lung and colon adenocarcinoma samples, collected 1–21 years before analysis, with tumour cellularity of 5%–60% [7]. They performed LowPass (shallow) WGS and OncoSeek panels and demonstrated a relationship between the number of cells recovered and NGS coverage uniformity, setting a 60‐cell‐threshold. In our study, we used a median of 51 cells (range 12–413) for sCNA analysis and obtained good‐quality data in 7/10 samples (70%). Importantly, the DEPArray_FFPE_QC kit predicted failure of NGS in 2/3 samples, suggesting that samples with DEPArray‐QC scores < 0.2 should not be further processed. As for the number of cells, we found no difference in the failure rate between libraries of ≤ 30 and > 30 cells (22% versus 28%), with only two libraries of ≤ 20 cells from one sample with high DEPArray‐QC failing. Our data suggest that a minimum of 30 cells should be used. However, the more cells input as DNA template, the higher the resolution of the data (Figure 3).

Importantly, the length of storage time of the FFPE blocks did not appear to correlate with the quality of the samples and NGS success. In fact, the two oldest samples (10 years) had the highest DEPArray‐QC scores. This finding is in contrast to several other studies [7, 22, 23], although those studies used samples > 10 years old.

One of the major factors affecting sCNA detection is tumour purity. In our study we used 50‐μm‐thick unselected FFPE sections from blocks taken during surgery or biopsy, contrasting with a previous DEPArray study, where 20/23 (87%) samples were 0.6 mm FFPE cores taken from representative tumour areas [7]. Cores have been used in pathology to enrich for carcinoma cells in low‐tumour‐cellularity samples. This approach is less laborious compared to laser‐guided microdissection but is still dependent on a histopathologist identifying the tumour‐enriched area. We managed to separate pure carcinoma populations with detected sCNA from tissues with tumour cellularities ranging from 10% to 85%, paving the way to study low‐tumour‐content samples that might otherwise give undetectable data using whole‐tissue‐based approaches. There was no correlation between the number of sCNA, TFx, and MAPD scores in those populations with tumour cellularity (data not shown), in contrast with other BC studies [22, 24], implying that DEPArray sorting removes this bias.

An important finding of our study was the overperformance of the DEPArray workflow compared to the workflow using tissue cores taken from representative tumour areas for samples with low tumour cellularity. The most striking difference was in losses (one copy versus two copies at baseline) but also in some gains (three copies versus the two copies at baseline). This agrees with other studies that have shown that borderline alterations can be false‐negative sCNA calls, particularly in low‐tumour‐fraction specimens [7, 24, 25, 26]. Of course, we should be cautious interpreting these data, since although we used the same bioinformatic analysis, the libraries were prepared/run with different kits/NGS platforms (Illumina_NextSeq_2000 versus Ion_Torrent_S5 for the DEPArray and coring workflow, respectively). In addition, when we compared the DEPArray workflow with microdissection for the two HGSC FFPE samples, we found similar tumour fractions, although with much higher background noise using the DEPArray protocol. This could be attributed to the fact that we used only ~25 carcinoma cells for those analyses as opposed to some tens of thousands of cells used in the microdissection protocol.

Within the DEPArray™ workflow, a ploidy estimation of the recovered carcinoma cells is available, providing an input to the low‐pass sCNA algorithm, in order to appropriately set the baseline and facilitate interpretation of the CN profiles [7, 19]. In our study, we detected aneuploid and pseudo‐diploid populations, and the DI estimated the ploidy correctly (in agreement with the best ploidy fitting of the ichorCNA algorithm) in 7/12 carcinoma populations (58%), suggesting that it can be useful but should be used with caution. The main reason is that the low number of cells used did not provide adequate DNA input, preventing the appearance of clear peaks in the DAPI integral intensity histogram used for the calculation of DEPArray‐DI. Accurate estimation of ploidy is important, as shown in BC [27] and HGSC [28].

Characterisation of sCNA in cancer is essential, since many human cancer types, including breast and ovarian, are CNA‐driven (C‐class) rather that mutation‐driven (M‐class), with sCNA playing a key role in determining their expression profiles [29]. When these sCNA affect driver‐genes, they influence tumour development and disease outcomes. In our study, we focused on 93 protein‐coding cancer genes with probable driver aberrations, identified by whole‐genome analysis of 560 breast cancers [10]. The sCNA in some of those genes, such as *FGFR1*, *MYC, CCND1, TP53, PIK3CA, EGFR, ERBB2*, have been found to be have prognostic value [30, 31], associated with time to relapse [32], metastasis to the lymph node [33], response to neoadjuvant chemotherapy [34], and endocrine therapy resistance [35, 36]. Similarly, our findings demonstrate the presence of sCNA in genes that could have prognostic or predictive value; cases with *PIK3CA* gene amplification (Samples 2, 3, 11) could be treated with PI‐3 K/AKT inhibitors like Alpelisib [37, 38]. Cases with *RB* loss and/or *CDK6* amplification, (Samples 2 and 6) might be resistant to Palbociclib and other CDK4/6 inhibitors [39]. FGFR inhibitors, such as AZD4547, might be effective for cases with *FGFR* amplifications, (Sample 3) [40, 41]. Cases with *BRCA1/2* loss (Samples 2 and 6) might be sensitive to Olaparib or other poly‐(ADP‐ribose) and polymerase inhibitors [42]. We also detected *ESR1* loss in Samples 2 and 11, which may influence the response to anti‐oestrogen therapy [43, 44]. Another important finding was that in some ER + HER2‐ cases of mBC (Samples 2 and 3), we found *ERBB2* gain or amplification, respectively. This suggests that HER2 amplification might have been missed using conventional detection methods or that the tumour may have evolved through the course of the disease, as found in other studies [45, 46]. In any case, these findings could have provided a potential option for anti‐HER2 targeted treatment, particularly with the increased recognition of the HER2‐low phenotype (HER2 IHC 1+ or 2+ and FISH‐negative), where antibody drug conjugates, such as Trastuzumab deruxtecan are used [47]. Regarding the HGSC samples, *CCNE1*, *KRAS* amplifications, related to platinum chemotherapy resistance [48], were found in Sample 8 (both) and Sample 9 (only *CCNE1*). Sample 8 with *BRCA2* loss may benefit from a PARP inhibitor, while Sample 9 could have been treated with anti‐HER2 therapy.

We also used the OncoSeek panel in seven patients (five overlapping with LowPass‐WGS), using more cells (median, 152; range 41–432), as suggested by the EAT calculations. Compared to these calculations, we had sufficient cells in 80% of the cell selections. We obtained NGS data in 6/7 samples, with a library failure rate of 13%. In four samples that we compared with OncoSeek, there was 100% concordance between amplifications called as significant in the OncoSeek panel and the LowPass‐WGS, similar to previous studies [7]. While the MSBiosuite OncoSeek analysis does not call gains and losses, we found good concordance in 50% of the samples. The discordance observed for two samples is likely due to the different algorithms used, the fact that MSBiosuite uses stromal cells as controls, while ichorCNA is control‐free, and also the LowPass‐WGS data for these samples were noisy. For mutation calling, all samples had somatic but also germline (data not shown) mutations, including some low‐VAF somatic variants that would have been missed in an unsorted sample. The high frequency of *TP53* mutations (in 3/4 BC and 2/2 HGSC samples) is in agreement with previous studies [10, 28], while other common drivers, such as *EGFR, PTEN, CDH1, CDKN2A, APC, ATM*, and *MAP2K1*, were found to be mutated in our study.

This technology and workflow may also be important for patients with lower or undetectable, in the case of primary cancer patients, circulating tumour cell (CTC) counts. Studies from our group and others [9, 45] have shown that it is possible to sequence single CTCs. However, some mBC patients do not have detectable CTCs, despite having widespread metastatic disease, and rely on metastatic biopsies for molecular characterisation. The increasing use of neoadjuvant chemotherapy in many cancers means that frequently there are only small core biopsies from the primary tumour for genomic analysis, for which the technology described here may be useful.

The major limitations of our study are its small scale and the low representation of various tissues and BC subtypes. Since the robustness of genomic data is dependent on the sample quality, the small number of samples and particularly of those compared using different methodologies did not allow us to reach a certain conclusion. Nevertheless, this proof‐of‐concept study demonstrates that the workflow described, utilising digital cell separation from small FFPE samples having been stored for long periods, allows accurate molecular analysis even in specimens with low tumour cellularity. This approach offers the potential to identify actionable targets for guiding treatment options in cancer.

## Author contributions statement

GN, EW, AR and DF‐G performed the DEPArray experiments (FFPE sample preparation, staining, and quality‐control check, DEPArray cell sorting). FM, OA, RC and NM performed the library preparation and NGS experiments. MKW and GN performed the bioinformatic analysis. EW, AR, RCC, KLTG, RF and LK assessed, selected, and provided the clinical samples. KP and RCA performed the FFPE coring/Ion Torrent workflow experiments and the related data analysis. ZC and DPE performed the laser microdissection/NGS workflow experiments and the related data analysis. JB supervised and supported the DEPArray experiments. IP cut sections of the FFPE tissue block. GN wrote the article, and analysed and interpreted the data. JAS, RCC, IAM and EW edited the article and contributed to data analysis and interpretation. GN, RCC and JAS designed the study. All authors revised and approved the article.

## Supporting information


**Supplementary materials** **and methods**
**Figure S1**. DEPArray report demonstrating the gating strategy for selecting carcinoma and stromal populations
**Figure S2**. Representative copy number (CN) profiles of samples with MAPD > 0.3 that failed next‐generation sequencing (NGS) QC metrics
**Figure S3**. Ploidy fitting performed by ichorCNA for Sample 2 comparing ploidies 2, 3, and 4 (the one close to the suggested by the DNA Index (DI) as calculated by DEPArray)
**Figure S4**. Copy number (CN) profiles of stromal and carcinoma populations for Sample 3
**Figure S5**. Copy number (CN) profiles of stromal and carcinoma populations of different DNA Indexes for Sample 6
**Figure S6**. Copy number (CN) profiles of stromal and carcinoma populations for Sample 8 (S6A) and Sample 9 (S6B)
**Figure S7**. Comparison of the DEPArray copy number (CN) profiles with those derived from the same tissue samples (samples 8, 9) after microdissection using two different packages: QDNAseq and ichorCNA
**Figure S8**. The 19 genes for somatic copy number alterations (sCNA) analysis included in the OncoSeek panel
**Table S1**. Summary of samples included in the study
**Table S2**. Effective amplifiable template (EAT) minimum for each downstream application, depending on different QC scores
**Table S3**. Comparison of somatic copy number alterations (sCNA) called by LowPass WGS and the OncoSeek panel in the four samples run with both technologies

**Table S4.** Mutation analysis performed in six samples for 56 genes included in the OncoSeek panel (provided as separate Excel file)

## Data Availability

The sequencing data (fastq files) are publicly available via the European Genome‐phenome Archive at the European Bioinformatics Institute (https://ega-archive.org) with accession numbers EGAS50000001327 (https://ega-archive.org/studies/EGAS50000001327) and EGAS50000001328 (https://ega-archive.org/studies/EGAS50000001328).
