# Supplementary material for: Identification of actionable targets using DEPArray‐based sorting of pure carcinoma and stromal populations from formalin‐fixed paraffin‐embedded tissues followed by shallow whole‐genome sequencing
Source: J Pathol. 2025 Oct 27;268(1):13–26. doi: 10.1002/path.6469 (PMC12699240; doi:10.1002/path.6469)
Supplement: Supplementary file 1 — Supplementary materials and methods Figure S1. DEPArray report demonstrating the gating strategy for selecting carcinoma and stromal populations Figure S2. Representative copy number (CN) profiles of samples with MAPD > 0.3 that failed next‐generation sequencing (NGS) QC metrics Figure S3. Ploidy fitting performed by ichorCNA for Sample 2 comparing ploidies 2, 3, and 4 (the one close to the suggested by the DNA Index (DI) as calculated by DEPArray) Figure S4. Copy number (CN) profiles of stromal and carcinoma populations for Sample 3 Figure S5. Copy number (CN) profiles of stromal and carcinoma populations of different DNA Indexes for Sample 6 Figure S6. Copy number (CN) profiles of stromal and carcinoma populations for Sample 8 (S6A) and Sample 9 (S6B) Figure S7. Comparison of the DEPArray copy number (CN) profiles with those derived from the same tissue samples (samples 8, 9) after microdissection using two different packages: QDNAseq and ichorCNA Figure S8. The 19 genes for somatic copy number alterations (sCNA) analysis included in the OncoSeek panel Table S1. Summary of samples included in the study Table S2. Effective amplifiable template (EAT) minimum for each downstream application, depending on different QC scores Table S3. Comparison of somatic copy number alterations (sCNA) called by LowPass WGS and the OncoSeek panel in the four samples run with both technologies [file PATH-268-13-s001.docx]

**Identification of actionable targets using DEPArray-based sorting of pure carcinoma and stromal populations from-formalin-fixed paraffin-embedded tissues followed by shallow whole-genome sequencing**

G Nteliopoulos *et al. J Pathol* <https://doi.org/10.1002/path.6469>

**Supplementary materials and methods**

**Supplementary Figures S1–S8**

**Supplementary Tables S1–S3**

**Supplementary Table S4 is provided in a separate Excel file**

**Reference numbers refer to the main text list**

**Supplementary materials and methods**

**Sample preparation of FFPE sections**

Tissue sections were sealed in a nylon biopsy bag inside a 50-ml conical tube, deparaffinized with the modification of using Histo-Clear (Agar Scientific, Rotherham, UK) instead of xylene (by three sequential 10 min incubations), and rehydrated using decreasing concentrations of ethanol. To prepare a cell suspension, tissue sections were washed with deionized water, followed by a heat-induced antigen retrieval step. Sections were incubated in antigen retrieval buffer (Menarini Silicon Biosystems (MSB), Bologna, Italy) containing citric acid monohydrate (pH 6.4) for 5 min at room temperature (RT) and heat-treated in the same prewarmed buffer for 1 h at 80 °C. After cooling at RT, the sections were washed 3 times in RPMI medium (Thermo Fisher Scientific, Paisley, UK), incubated for 5 min, and then subjected to tissue digestion by incubation at 37 °C in enzymatic dissociation solution containing proteinase, *Bacillus polymyxa* neutral protease and collagenase (MSB) for up to 45 min. The reaction was stopped by placing the sample tube on ice. After the filtration and washing steps, cells were stained with 50 μg/ml Hoechst 33342 (Thermo Fisher Scientific, Altrincham, UK) and counted using a Countess® II FL Automated Cell Counter (Thermo Fisher Scientific).

The cell pellet was then resuspended in a working solution buffer containing blocking reagent (BSA) and permeabilization reagent (Tween-20). A total of 500,000 cells were used for immunofluorescent labelling stained with DAPI (Thermo Fisher Scientific) for nuclear staining, anti-cytokeratin (CK) A (pan-cytokeratin monoclonal antibody raised against cytokeratin 4, 5, 6, 8, 10, 13, 18), anti-cytokeratin B (broad spectrum anti-keratin monoclonal antibody reacting with all members of the basic subfamily and the 56.5, 50, 50’, 48, and 40 kDa keratins of the acidic subfamily) and anti-vimentin (VIM) antibody detecting vimentin in the cytoplasm of cells of mesenchymal origin. For staining, 100 μl of primary monoclonal antibody mixture containing anti-keratin MNF116, IgG1 (DAKO, Glostrup, Denmark) (final concentration, 3.2 μg/ml); anti-keratin AE1/AE3, IgG1 (Millipore–Chemicon, Burlington, MA, USA) (final concentration, 10 μg/ml); and anti-vimentin 3B4, IgG2a (DAKO) (final concentration, 3.1 μg/ml) was used, with cells incubated in working solution for 30 min at 4°C. Primary antibodies were then visualized using fluorophore-conjugated secondary antibodies; Alexa Fluor®488 Goat Anti-Mouse IgG1 (Thermo Fisher Scientific) (final concentration, 2.5 μg/ml for anti-cytokeratin) and Alexa Fluor®647 Goat Anti-Mouse IgG2a (Thermo Fisher Scientific) (final concentration, 2.5 μg/ml for anti-vimentin antibody). Cells were incubated with secondary antibody mix, also containing 10 μM DAPI (Sigma-Aldrich), in working solution for 30 min at 4 °C in the dark. The stained cells were counted using the Countess® II FL Automated Cell Counter (Thermo Fisher Scientific).

**Quality control of FFPE samples**

The DEPArray™ FFPE QC Kit (Menarini Silicon Biosystems (MSB), Bologna, Italy) was used to assess DNA integrity of the cell suspensions prior to DEPArray™ experiments and subsequent next-generation sequencing (NGS). An input sample of 1,500 unstained cells, obtained using the DEPArray™ FFPE SamplePrep Kit (MSB), were pelleted and lysed following the manufacturer’s protocol. Quality assessment was performed using a real-time qPCR-based assay, using two different primer pairs, which produced amplicons of 54 bp and 132 bp. The quality of DNA was calculated as the ratio between the relative amounts of long and short amplicons. Intact, highly preserved DNA showed equal prevalence of the two amplicons (ratio close to 1), whereas a highly fragmented DNA showed a higher prevalence of the shorter 54 bp amplicon (ratio close to zero). This ratio provides the QC score indicating what percentage of the fragmented DNA molecules will be effectively available for downstream analysis. The QC score, combined with two objective parameters obtained through DEPArray sorting (the number of cells isolated and their ploidy level), enables calculation of the Effectively Amplifiable Template (EAT) in the sample (EAT = QC score × number of cells × ploidy). EAT has a predictive value for the outcome of the library preparation. Different downstream assays have application-specific minimum EAT requirements.

**DEPArray NxT preparation and running**

Before proceeding with the DEPArray™ NxT run, 80,000 stained cell suspension cells were washed and incubated in DEPArray buffer for fixed cells (MSB) for at least 16 h. A total of 24,000 cells in 12 μl, was loaded to the DEPArray™ NxT cartridge, following the manufacturer’s protocol. CellBrowser™ analysis software, integrated into the DEPArray ™ system (MSB), was used to view and select cells according to multiple criteria, including qualitative and quantitative marker assessment and cell morphology. After scanning the main chamber for the presence of single cells, the software applied an automated gating strategy for tumour and stromal cell identification, ploidy selection, and fine cell selection as follows: Out of all cells in the main chamber, only those trapped within the DEP-cage and were routable, were selected. In a scatterplot based on routable cells, CK-AF488 and Vim-AF647 were displayed and the two populations, CK+/VIM−/DAPI+ tumour cells and VIM+/CK−/DAPI+ stromal cells, were defined. The DNA index (DI) of the two cell populations was measured indirectly using the integral intensity of DAPI fluorescence, which is in stoichiometric relationship to the cellular DNA content. For DI determination for various cell populations, the x-axis position of the peaks was measured in the related integral intensity DAPI histogram. The diploid stromal cells served as the normal DNA reference to identify pseudo-diploid and aneuploid carcinoma cell fractions. Next, clusters of two or three cells and clumps were excluded and only single cells with the desired fluorescence intensity and cell morphology were automatically selected. The desired number of cells per pool were calculated according to the EAT (as shown in the section ‘Quality control of FFPE samples’ of supplementary materials and methods), and the pools were recovered, and volume reduction performed following the manufacturer’s protocol (MSB). The isolated cells were stored at −20 °C for later downstream analysis.

**Whole-genome low-pass whole-genome sequencing (WGS) for somatic copy number alterations (sCNA) analysis**

Low Tris-EDTA (TE) buffer (Swift Biosciences / Integrated DNA Technologies, Leuven
Belgium; 46 μl) was added to the tube with the lysed cells. The sample was then fragmentated using the Covaris M220 instrument (Covaris, Brighton, UK) for 3 min and 52 s (pick power: 50, duty factor: 20, cycles/burst: 200) to obtain a 150–200 bp fragment size. Libraries were prepared using an Accel-NGS® 2S PCR-Free DNA Library kit (Swift Biosciences / Integrated DNA Technologies) following the manufacturer’s protocol. A 20 µl sample of the prepared library was amplified as follows: 6 μM of amplicon PCR forward primer (5′-AATGATACGGCGACCACCGAGATC-3′), 6 μM of amplicon PCR reverse primer (5′-CAAGCAGAAGACGGCATACGA-3′), and 2× KAPA HiFi HotStart Ready Mix (Kapa Biosystems/Roche, Burgess Hill, UK). The PCR cycling conditions were 98 °C initial denaturation for 45 sec, followed by 16 cycles and 15 cycles for ~100 cells and ~300 cells, respectively, at 98 °C for 15 sec, 60 °C for 30 sec, and 72 °C for 1 min, and a final extension at 72 °C for 1 min. The products were cleaned up with 0.75× Agencourt AMPure XP beads (Beckman Coulter, Amersham, UK) following the manufacturer’s protocol, and eluted in 20 μl low TE (Swift Biosciences / Integrated DNA Technologies). Libraries were normalized and pooled to 4 nM based on qPCR quantification. Pooled samples were denatured and diluted to a final concentration of 12 pM. All samples were multiplexed and sequenced on a NextSeq 2000 system (Illumina, Cambridge, UK), using 2 × 100 bp paired-end sequencing.

The fastq paired-end reads were aligned to the hg19 reference using BWA-MEM [8], and WIG files were created from the resulting BAM files using HMMcopy readcounter (<https://github.com/shahcompbio/hmmcopy_utils>, 30/07/2025) with bin sizes of 0.5 MB and a stringent mapping quality filter of 20. Somatic copy number alterations (sCNA) analysis was performed using IchorCNA v0.2.0 (<https://github.com/broadinstitute/ichorCNA>, 30/07/2025) applying default settings and no baseline (without control sample) to predict sample tumour fraction (TFx) and CN counts [9]. Read counts were corrected for GC content and mappability (using the uniqMatch option), and the main ploidy level was estimated for each library based on best-fitting profiles based on the CN levels. We inputted into ichorCNA the ploidy as calculated by the DI and then gave the software the option to select the best fit from diploid up to the ploidy value we provided, choosing the ploidy it considered the best fit in that range. Quality control metrics (as defined in the manufacturer’s instructions) required sequencing data to exceed >100,000 productive reads, with a median absolute pairwise difference (MAPD) <0.3, which provides a measure of read coverage noise. Actionable amplification and deletion analysis to intersect CN variations with 93 breast cancer related genes [10] was performed using bedtools (v2.31.0) [11]. CN states were classified as loss or hemizygous deletion (HETD, 1 copy), copy neutral (NEUT, 2 copies), copy gain (GAIN, 3 copies), amplification (AMP, 4 copies), and high-level amplification (HLAMP, 5–7 copies).

**OncoSeek panel**

Reagents were added to the lysed cells to prepare the DEPArray™ OncoSeek libraries (MSB). Each DEPArray™ OncoSeek library was diluted 1:10,000, and then quantified in triplicate by qPCR using the KAPA Library Quantification Kit (Hoffmann-La Roche, Basel, Switzerland) following the manufacturer’s protocol. Quantification was then adjusted for an average library size of 243 bp. All libraries were pooled, and NGS performed using an Illumina NextSeq 2000 system, using 150 bp paired-end sequencing, following the manufacturer’s protocol (Illumina). The fastq paired-end reads were inputted in the cloud-based MSBiosuite (MSB) and bioinformatic analysis was performed. fastq paired-end reads were trimmed using Cutadapt (<https://cutadapt.readthedocs.io/en/stable>, 30/07/2025) following the manufacturer’s protocol to remove synthetic primers from overlapping amplicons. The trimmed reads were aligned to the human reference genome (hg19) using the BWA software [1]. The alignment and coverage statistics were obtained using SAMtools (<https://github.com/samtools/samtools>, 30/07/25) and BEDTools [2] packages. After filtering to discard the partial, poorly aligned, and unmapped reads, variant calls were obtained using LoFreq software (<https://csb5.github.io/lofreq/>, 30/07/25). The resulting variants were annotated using the Ensembl Variant Effect Predictor (<https://www.ensembl.org/info/docs/tools/vep/index.html>, 30/07/25). All identified variants were filtered according to their presence/absence in public databases such as 1000 Genomes (<https://www.internationalgenome.org/>, 30/07/25), gnomAD (<https://gnomad.broadinstitute.org/>, 30/07/25), and COSMIC (<https://cancer.sanger.ac.uk/cosmic/>, 30/07/25), and their pathogenic effect.

CN alteration analysis of the OncoSeek data was performed using sorted populations as tests and a set of stromal cell pools from different samples as controls. For CN calling, the reads mapping to the target amplicons of the DEPArray™ OncoSeek panel were counted. Then, the read counts were normalized using the following two-step procedure: (1) Between-sample normalization using the total number of aligned reads and (2) Within-sample normalization using a LOWESS fitting of read counts with respect to the first component, explaining >90% of variation between regions in the control samples. Fold changes were computed by dividing the normalized counts in the test samples by the baseline, defined as the median value of the normalized counts per amplicon across the control samples. The final CN calls per gene were obtained by calculating the median fold changes in all gene-specific amplicons.

**
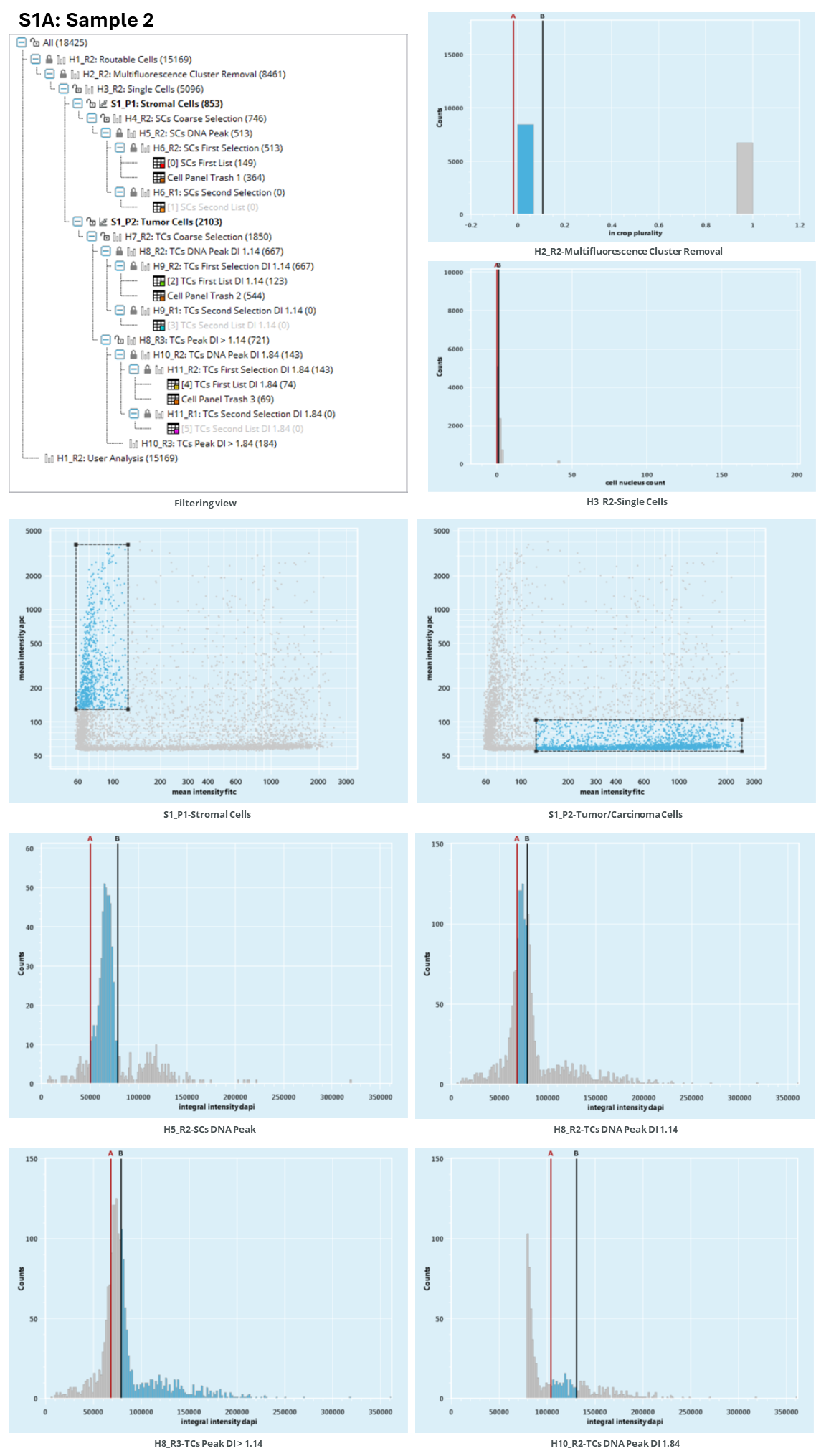
**

**
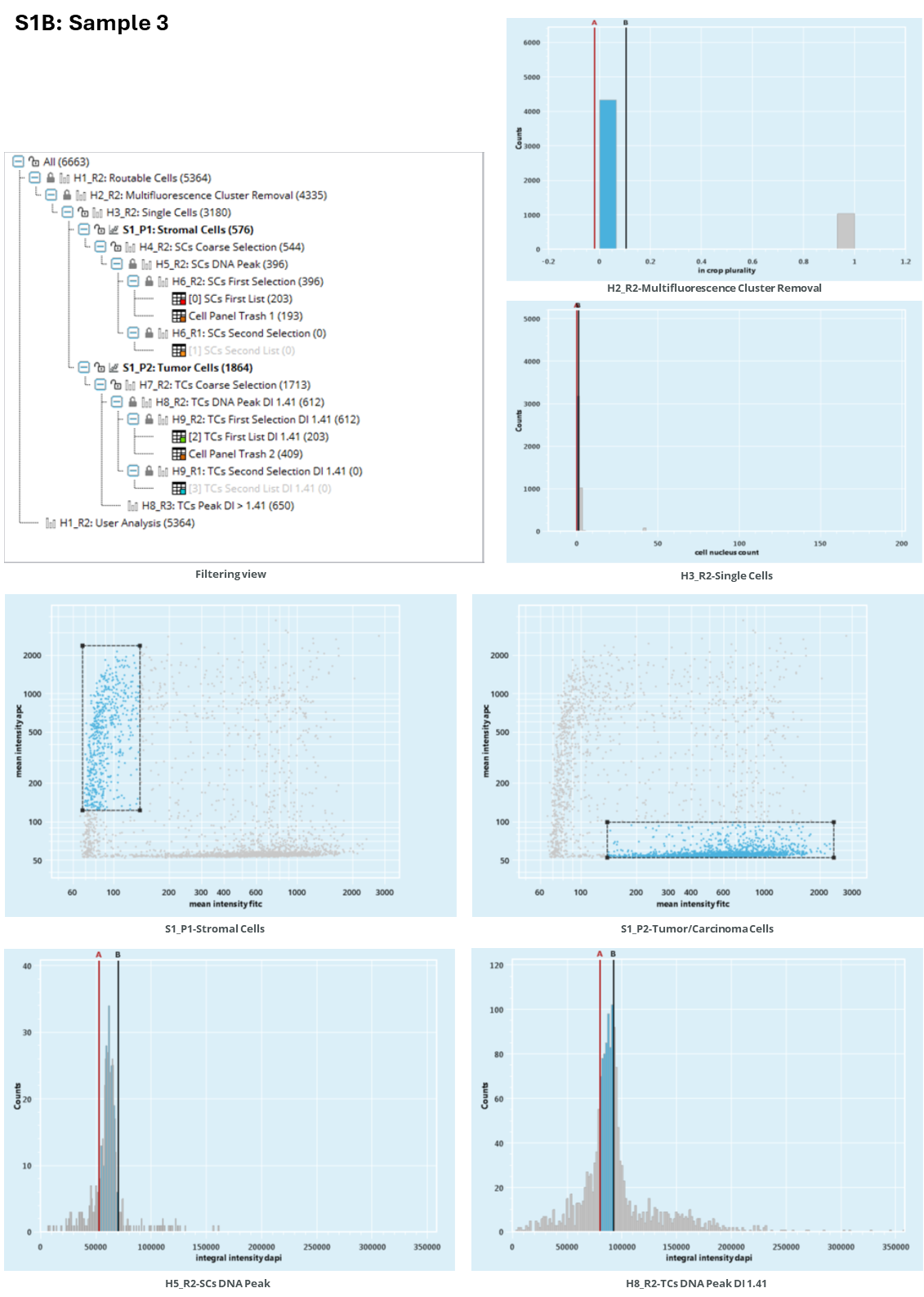
**

**
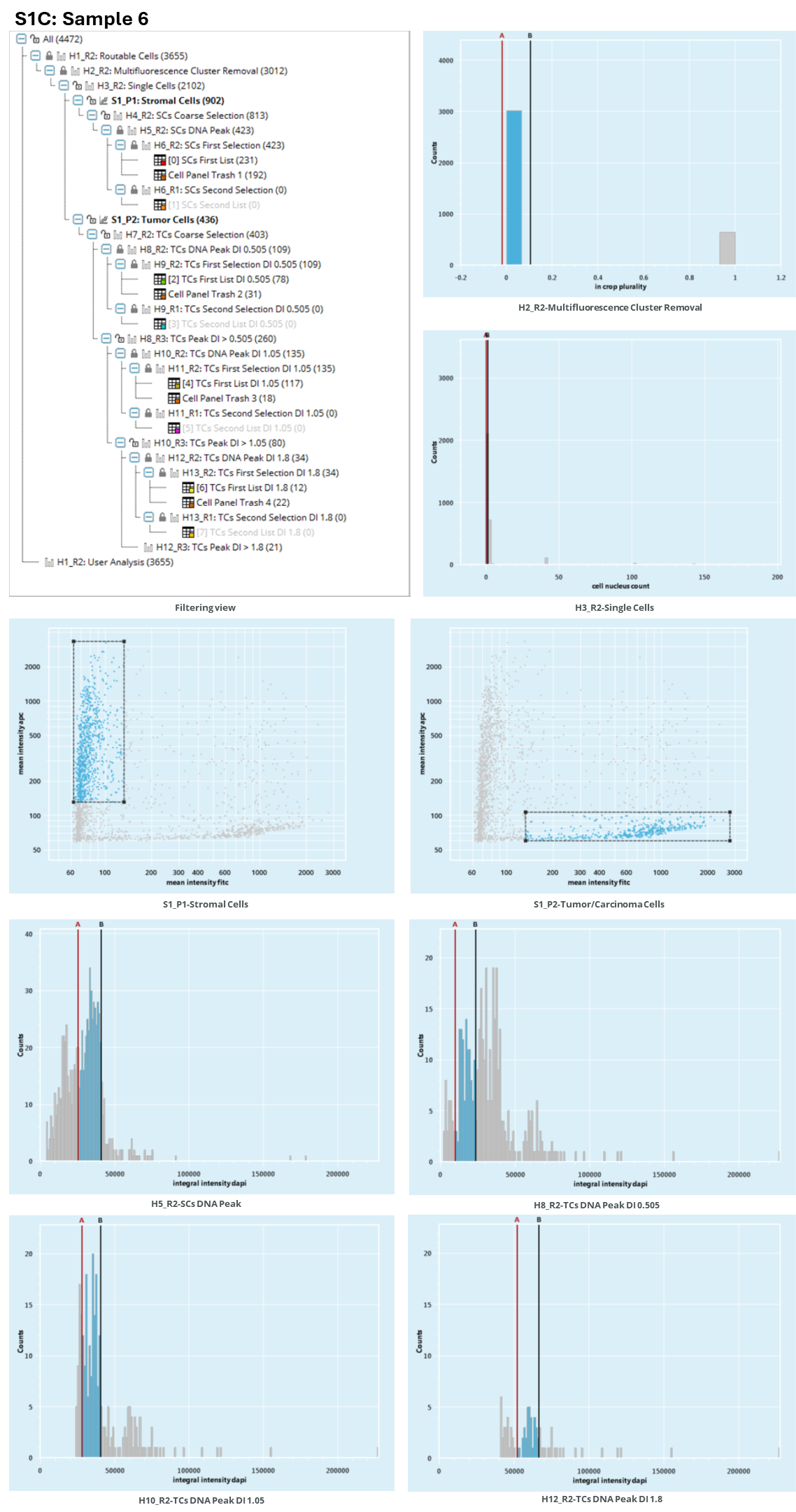
**

**
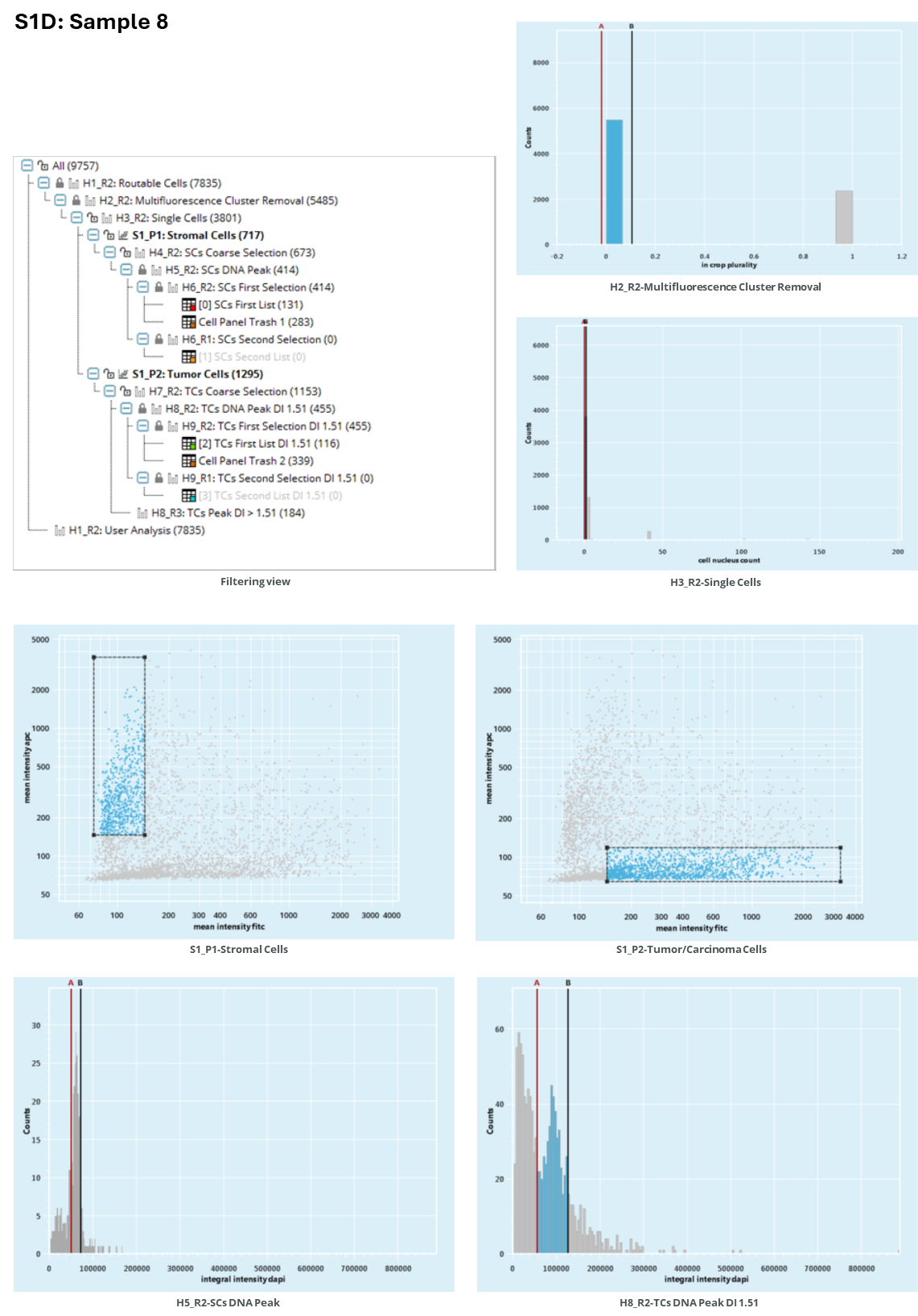
**

**
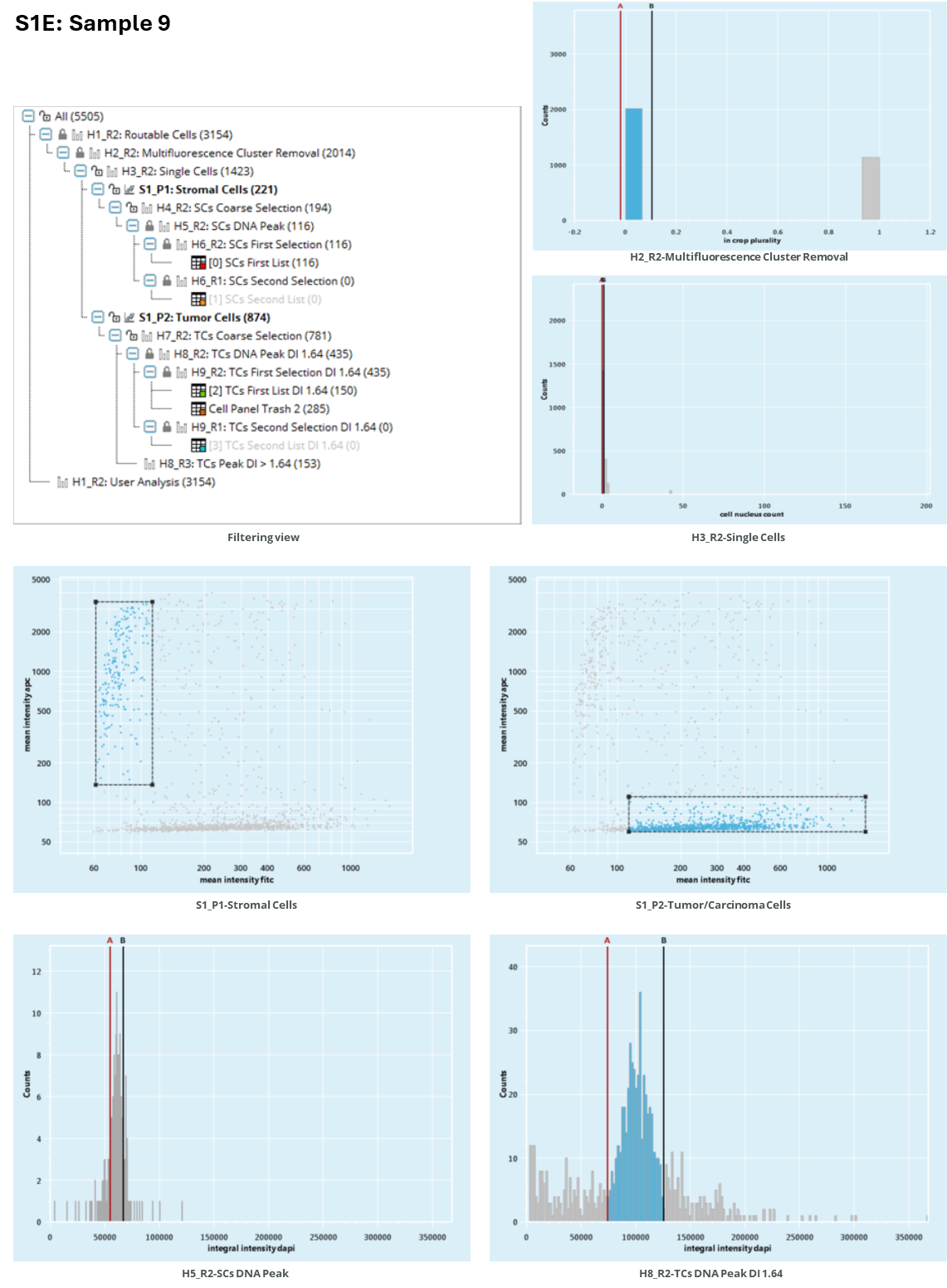
**

**
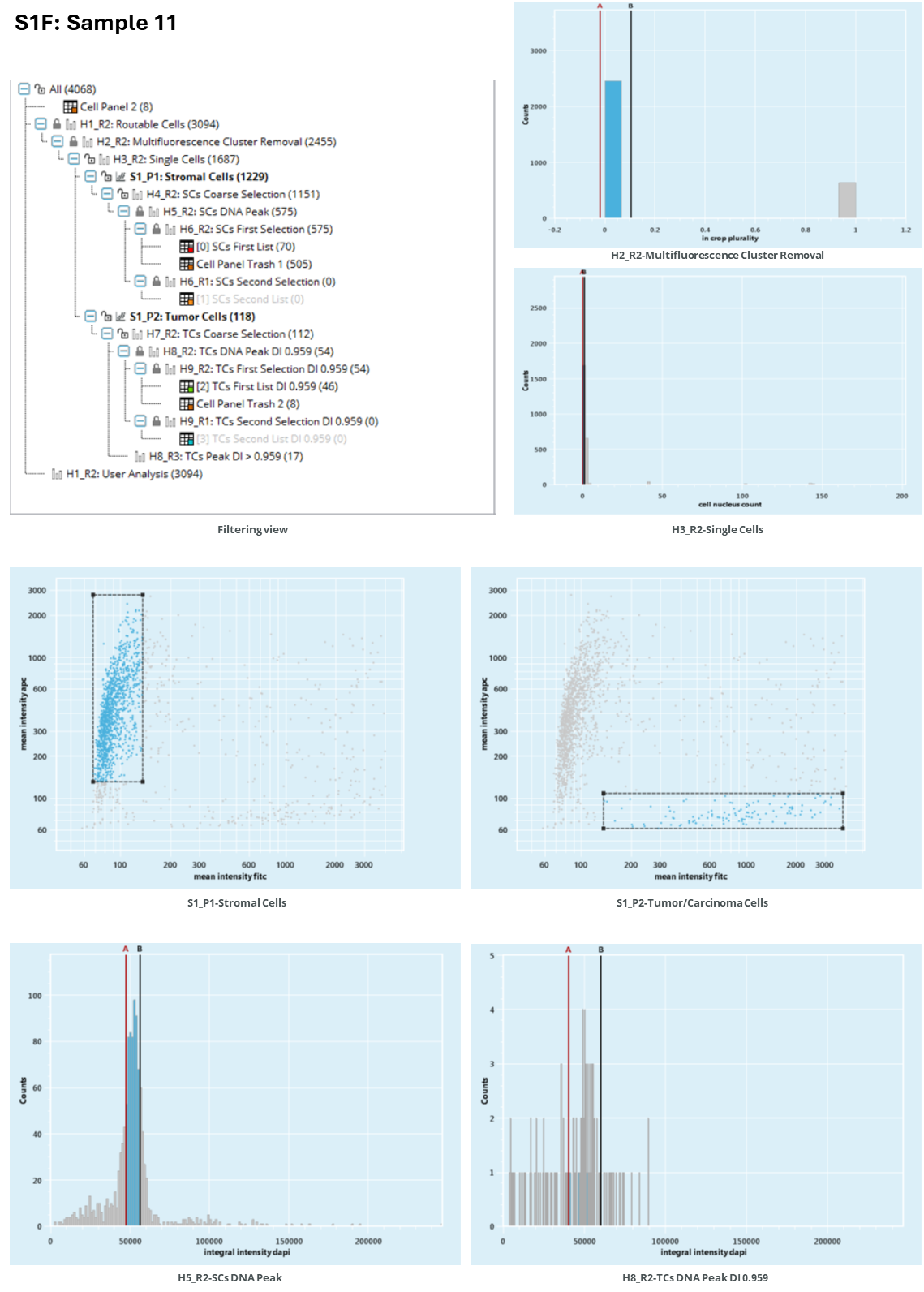
**

**
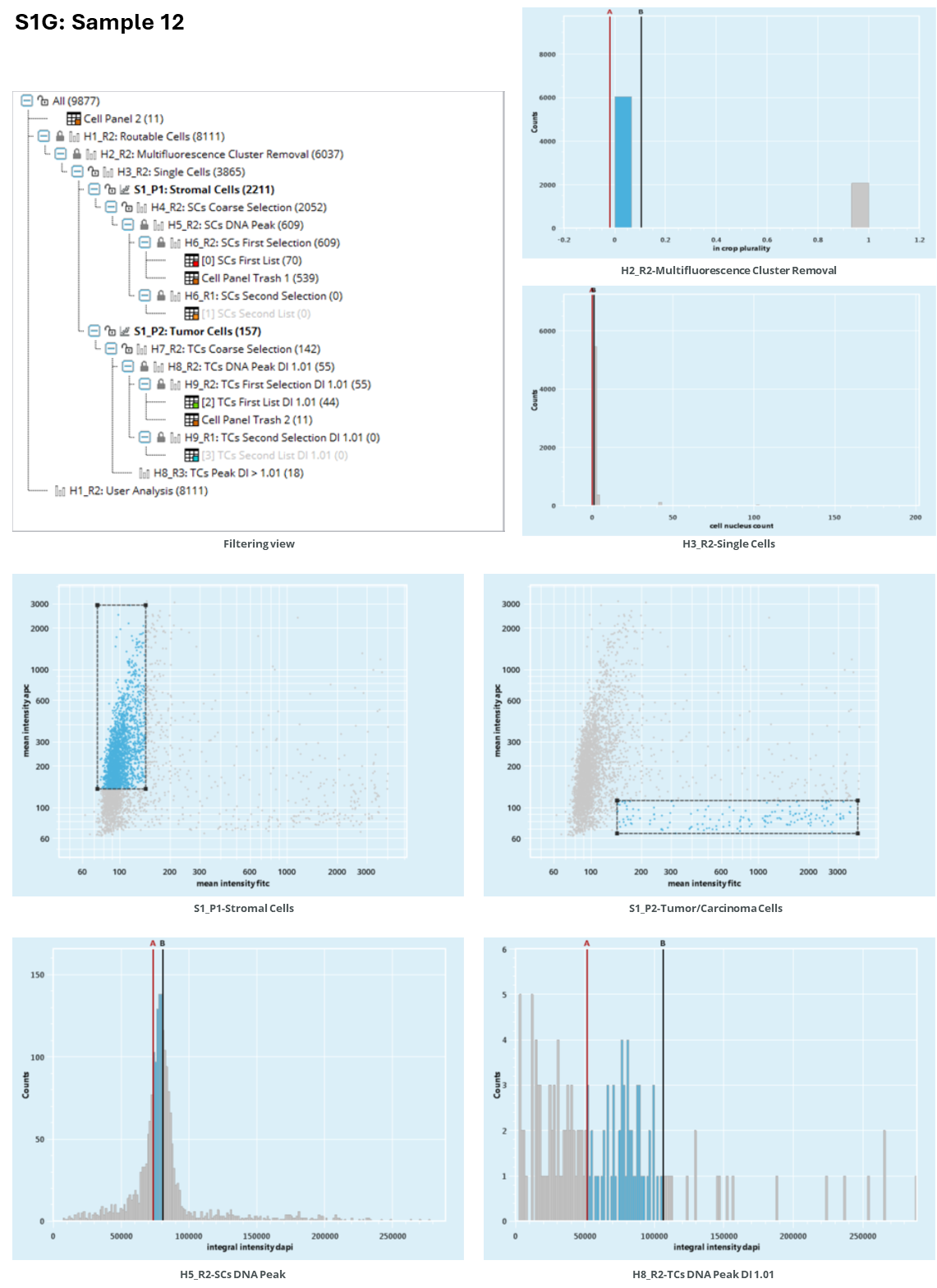
**

**
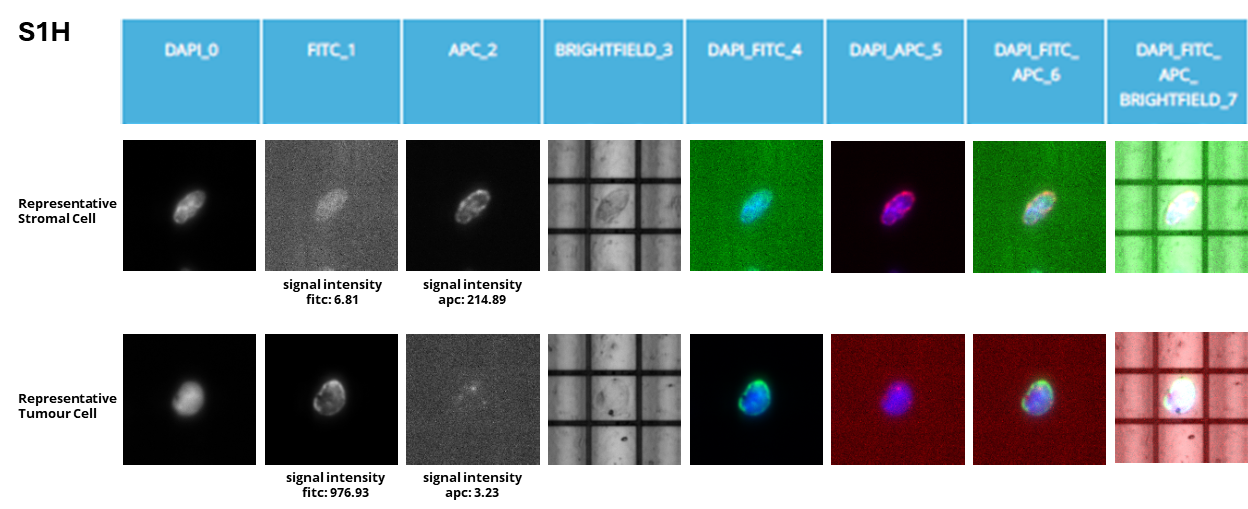
**

**Figure S1.** **DEPArray report demonstrating the gating strategy for selecting carcinoma and stromal populations.** (S1A–G) Filtering views show the number of cells available to select from each population after excluding nonroutable cells (not included in the DEP-cages) and cell clusters. For each subpopulation of single cells, the first selection (cells with the desired fluorescence intensity and cell morphology of an overall score >54) and the cell panel trash (overall score <54) are shown. Next the automated gating strategy is shown, including histograms for multi-fluorescence cluster removal and for single-cell selection, the dot plots displaying mean intensity of APC (*y*-axis), and mean intensity of FITC (x-axis) for selecting cytokeratin (CK)+/ vimentin (VIM)− carcinoma cells and CK−/VIM+ stromal cells. Histograms based on the integral intensity of DAPI used to calculate the DNA index (DI) In the carcinoma populations of samples 3 (S1B), 8, 9, 11, 12 (S1D-G), a single G1 peak is observed, whereas for sample 2 (S1A), two G1 peaks are observed and for sample 6 (S1C), three G1 peaks. In S1H, images of a representative CK−/VIM+ stromal and CK+/VIM− carcinoma cell are shown for the DAPI, FITC, APC and brightfield channels, and their overlays. For the FITC and APC channels, fluorescent intensities of the cell images are also depicted.


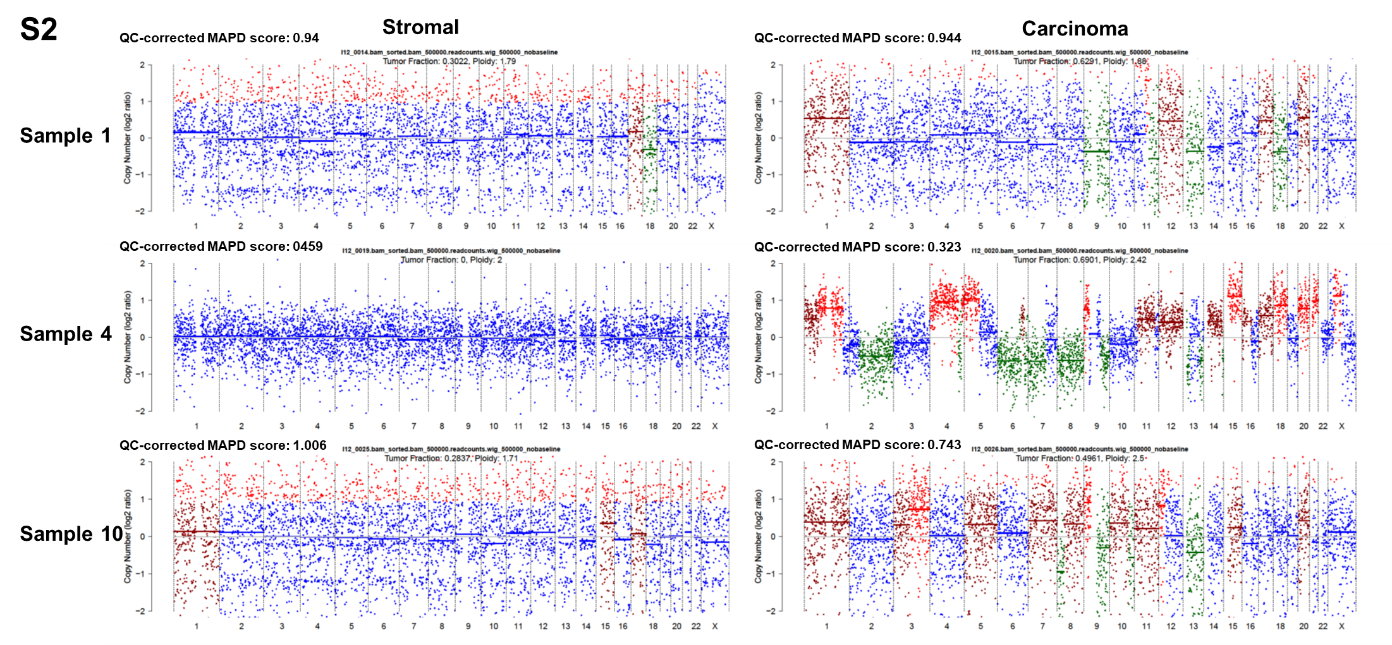


**Figure S2.** **Representative** **copy number profiles of samples with MAPD > 0.3 that failed next-generation sequencing (NGS) QC metrics.** The GC-corrected MAPD score, tumour fraction, and ploidy as calculated by ichorCNA, are shown for each copy number (CN) profile, using a 0.5 MB tile size parameter. Copy number losses (CN = 1) are highlighted green, gains (CN = 3) in dark red, amplifications (CN ≥ 4) in red, and baseline values in blue (CN = 2).


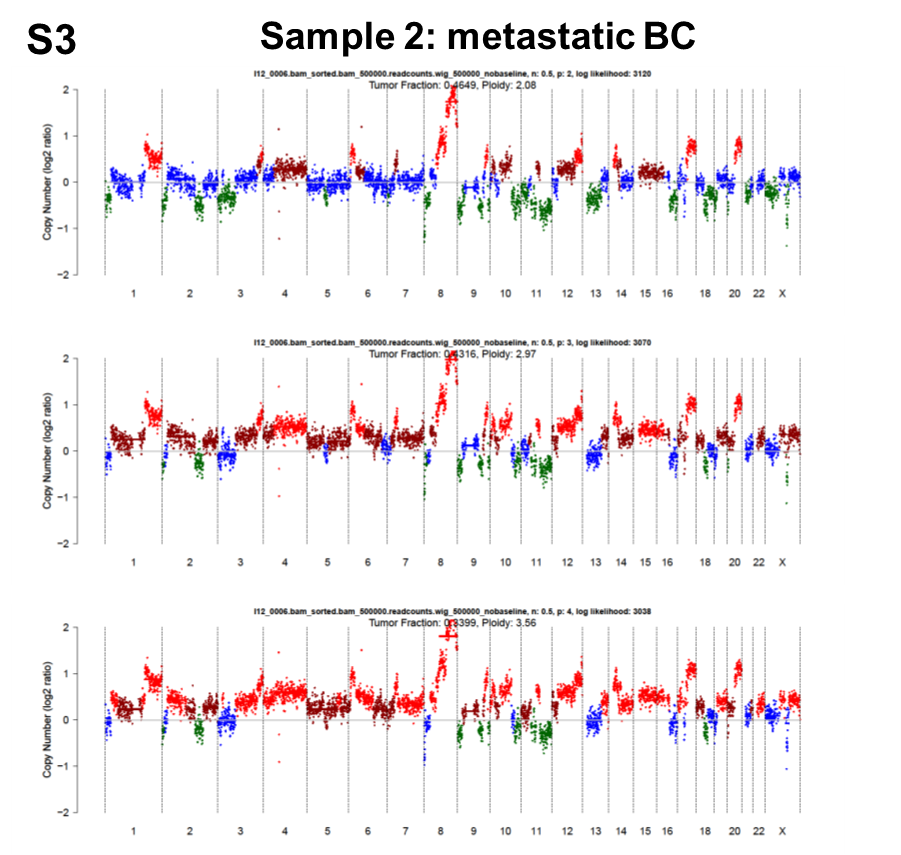


**Figure S3.** **Ploidy fitting performed by ichorCNA for Sample 2 comparing ploidies 2, 3, and 4 (the one close to the suggested by the DNA Index (DI) as calculated by DEPArray).** The tumour fraction and ploidy, calculated by ichorCNA, and the likelihood for each ploidy are shown in each copy number (CN) profile. Copy number losses (CN = 1) are highlighted green, gains (CN = 3) in dark red, amplifications (CN ≥ 4) in red, and baseline values in blue (CN = 2).


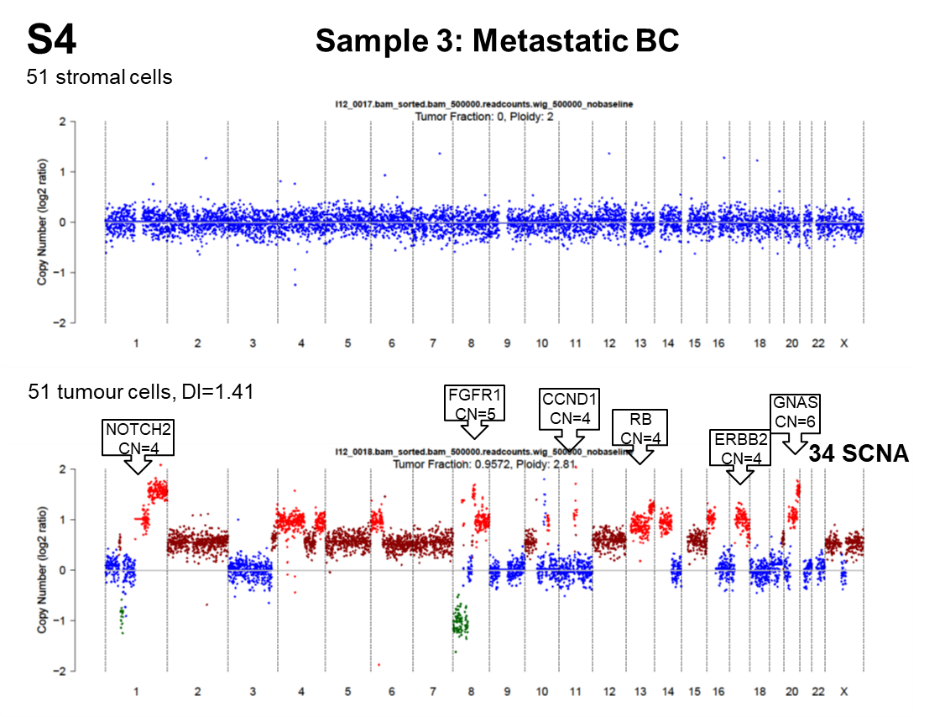


**Figure S4. Copy number (CN) profiles of stromal and carcinoma populations for Sample 3.** Tumour fraction and ploidy, calculated by ichorCNA, the number of cells, the DNA Index (DI), the number of somatic copy number alterations (sCNA) called, the breast cancer related genes overlapping with sCNA and the copy number (CN) for those genes versus the baseline are shown in each CN profile. CN losses (CN = 1) are highlighted green, CN gains (CN = 3) in dark red, amplifications (CN ≥ 4) in red, and baseline values in blue (CN = 2).


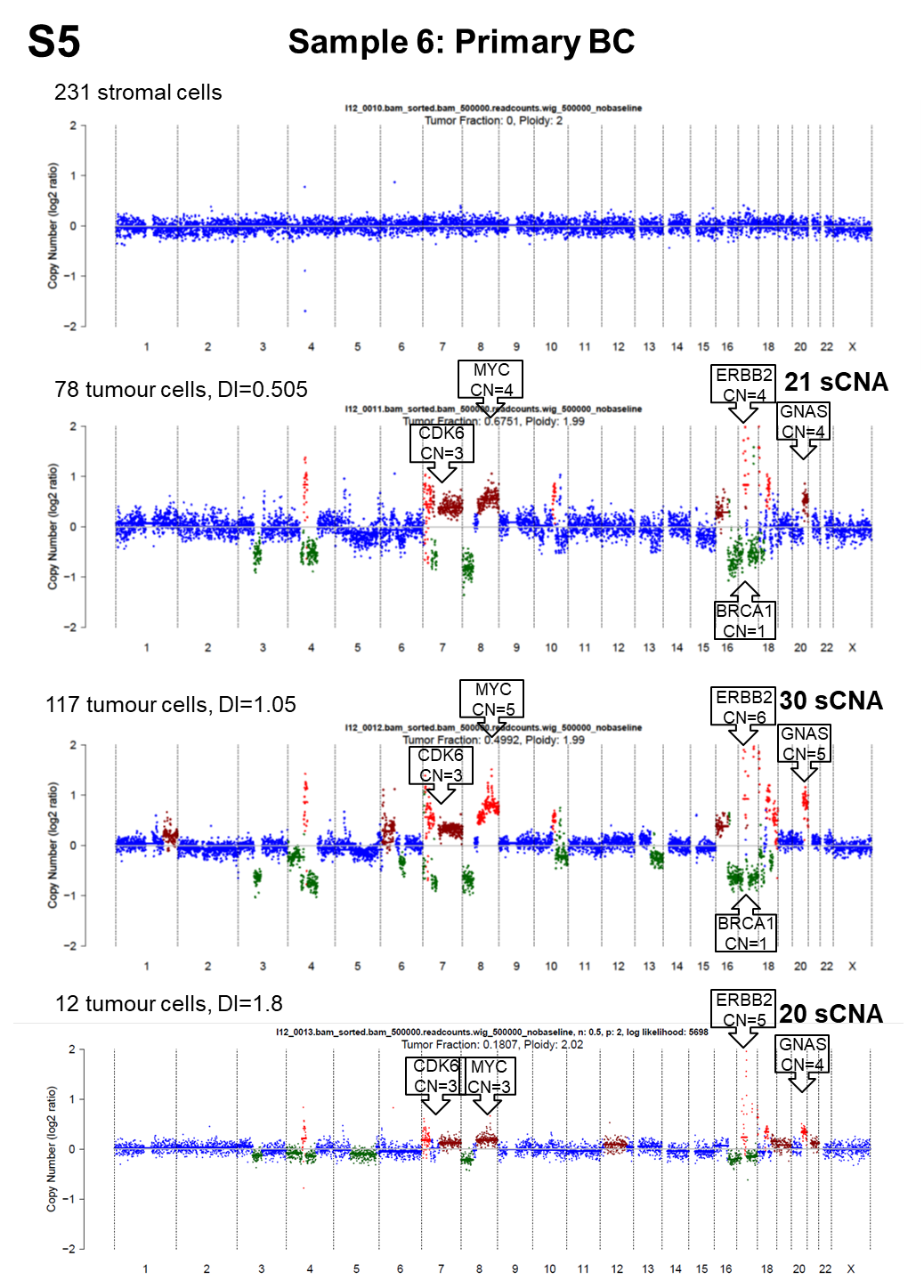


**Figure S5.** **Copy number (CN) profiles of stromal and carcinoma populations of different DNA indexes for Sample 6.** Tumour fraction and ploidy, calculated by ichorCNA, the number of cells, the DNA Index (DI), the number of somatic copy number alterations (sCNA) called, the breast cancer-related genes overlapping with sCNA and the copy number (CN) for those genes versus the baseline are shown in each profile. CN losses (CN = 1) are highlighted green, CN gains (CN = 3) in dark red, amplifications (CN ≥ 4) in red, and baseline values (CN = 2) in blue.


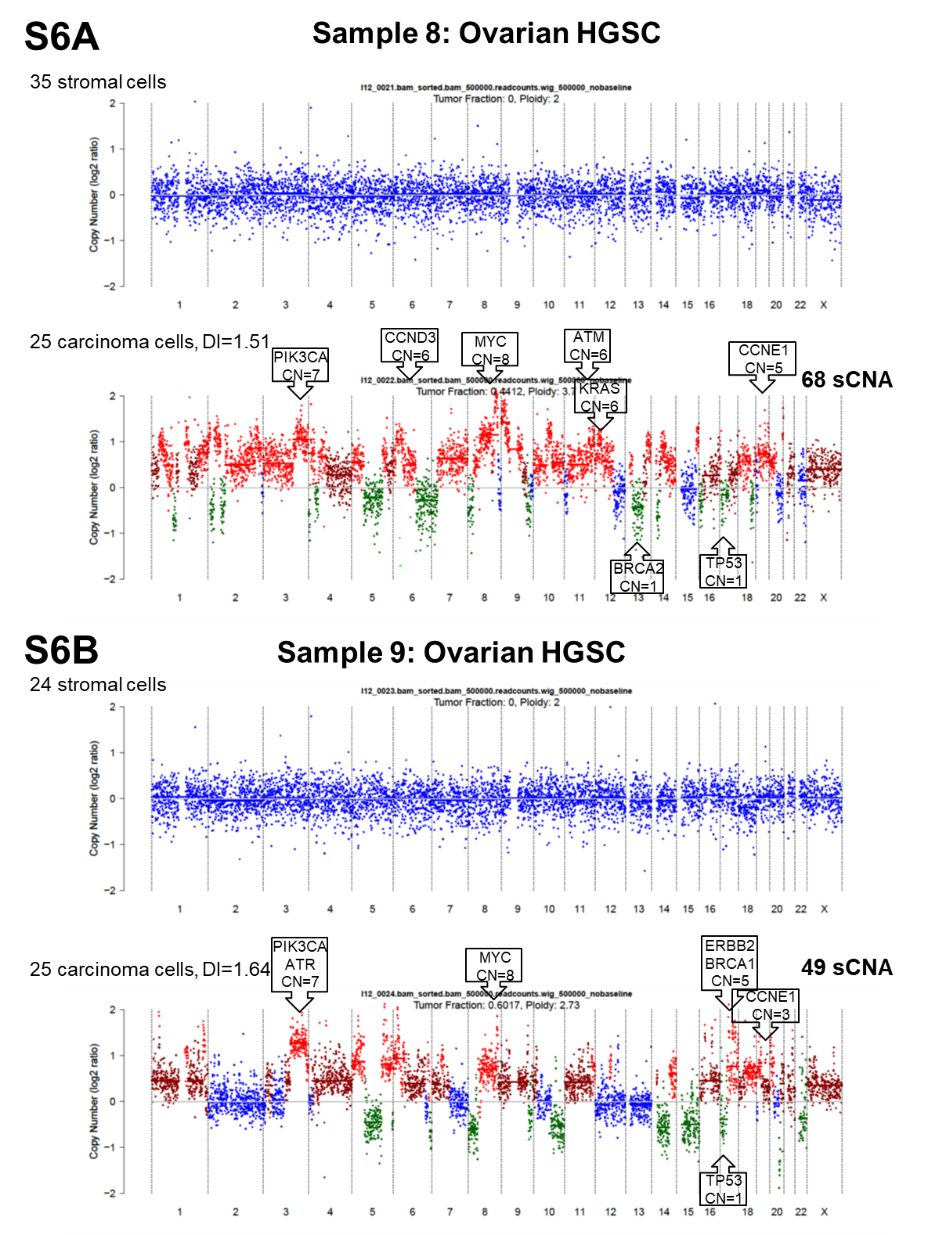


**Figure S6. Copy number (CN) profiles of stromal and carcinoma populations for Sample 8 (S6A) and Sample 9 (S6B).** Tumour fraction and ploidy, calculated by ichorCNA, the number of cells, the DNA Index (DI), the number of somatic copy number alterations (sCNA) called, the breast cancer-related genes overlapping with sCNA, and the copy number (CN) for those genes versus the baseline CN losses (CN = 1) are highlighted green, gains (CN = 3) in dark red, amplifications (CN ≥ 4) in red, and baseline values in blue (CN=2)


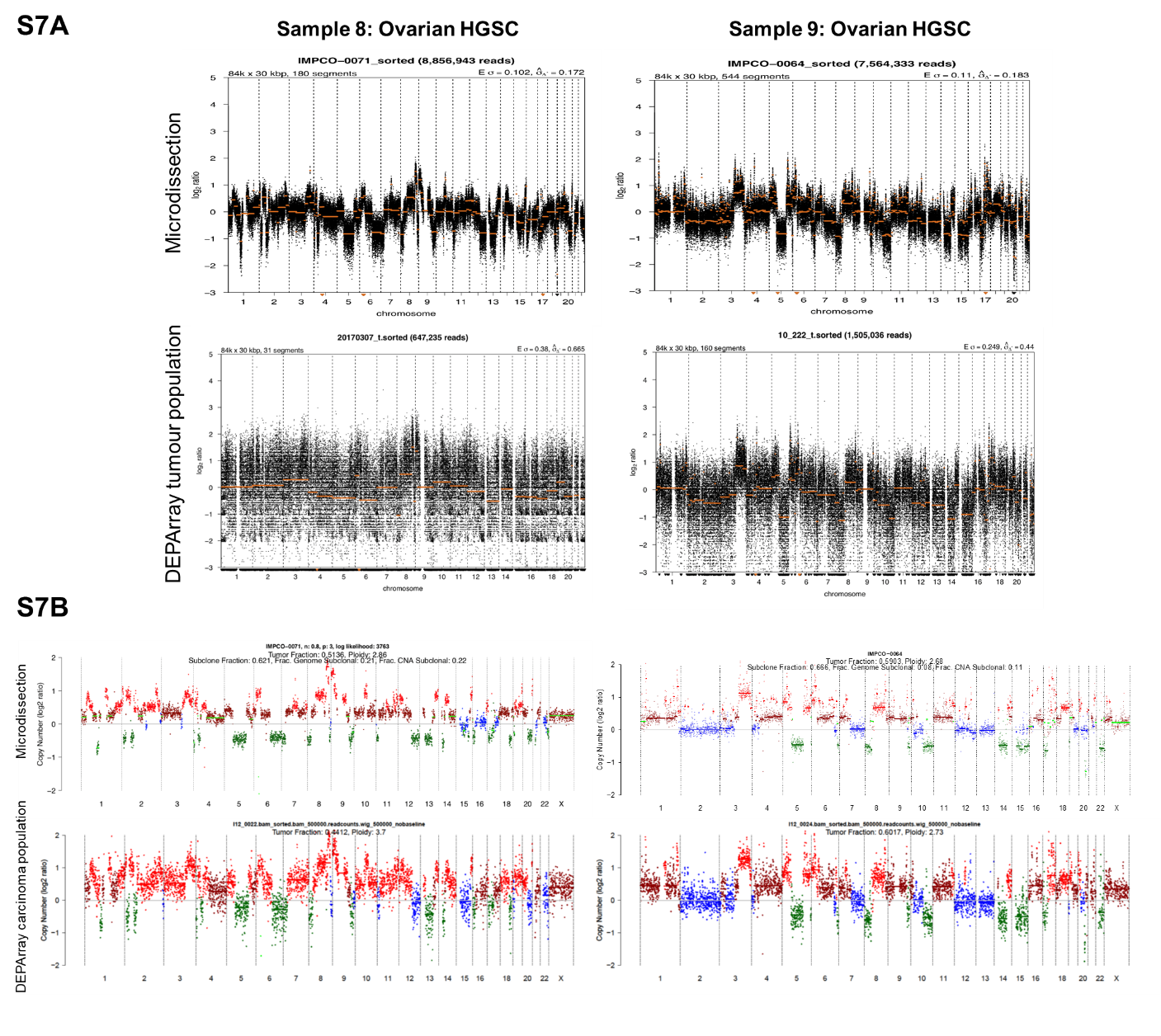


**Figure S7. Comparison of the DEPArray copy number (CN) profiles with those derived from the same tissue samples (samples 8, 9) after microdissection using two different packages: QDNAseq and ichorCNA.** The QDNAseq package, with a tile size of 30 kB (S7A), was used, where 𝐸𝜎 (expected standard deviation) and 𝜎Δ (change in standard deviation) are shown. The ichorCNA package, with a tile size of 500 kB, (S7B) was used to calculate tumour fraction and ploidy. Copy number (CN) losses (CN = 1) are highlighted green, gains (CN = 3) in dark red, amplifications (CN ≥ 4) in red, and baseline values in blue (CN = 2).

**Figure S8. The 19 genes for somatic copy number alterations (sCNA) analysis included in the OncoSeek panel.** Calls with a significant difference between stromal (red) and tumour (blue) cells are marked with a star.

**Table S1.** Summary of samples included in the study.

| **Sample** | **Tumour cellularity (%)** | **Tissue storage time (yrs)** | **Tissue** | **Sample ID** | **DEPArray single cells**  **(1^st^ + 2nd selection)** | **Cell type** | **DEPArray FFPE QC kit score** | **DEPArray DNA Index** | **DEPArray No. cells per library** | **No cells for downstream analysis** | **Downstream analysis** |
| --- | --- | --- | --- | --- | --- | --- | --- | --- | --- | --- | --- |
| s1 | 80 | 7 | Bone marrow | I12_0014 | 101 + 91 | stromal | 0.1 | 1 | 192* | 375 | LibPrep + LowPass WGS |
|  |  |  |  | I12_0015 | 319 + 203 | carcinoma | 0.1 | 1.25 | 325 | 300 | LibPrep + LowPass WGS |
|  |  |  |  | I12_0016 | 38 + 29 | carcinoma | 0.1 | 2.33 | 67* | 161 | LibPrep + LowPass WGS |
| s2 | 75 | 9 | ER^+^ PR^+^ HER2^−^ breast from surgery | I12_0001 | 149 + 364 | stromal | 0.56 | 1 | 31 | 27 | LibPrep + LowPass WGS |
|  |  |  |  | I12_0002 | 149 + 364 | stromal | 0.56 | 1 | 68 | 27 | LibPrep + LowPass WGS |
|  |  |  |  | I12_0003 | 123 + 544 | carcinoma | 0.56 | 1.14 | 22 | 23 | LibPrep + LowPass WGS |
|  |  |  |  | I12_0004 | 123 + 544 | carcinoma | 0.56 | 1.14 | 65 | 23 | LibPrep + LowPass WGS |
|  |  |  |  | I12_0005 | 74 + 69 | carcinoma | 0.56 | 1.84 | 16 | 15 | LibPrep + LowPass WGS |
|  |  |  |  | I12_0006 | 74 + 69 | carcinoma | 0.56 | 1.84 | 39 | 15 | LibPrep + LowPass WGS |
| s3 | 85 | 5 | ER^+^ HER2^−^ pleural biopsy | I12_0017 | 203 + 193 | stromal | 0.28 | 1 | 51* | 134 | LibPrep + LowPass WGS |
|  |  |  |  | I12_0018 | 203 + 409 | carcinoma | 0.28 | 1.44 | 51* | 93 | LibPrep + LowPass WGS |
|  |  |  |  | I14_0001 | 203 + 193 | stromal | 0.28 | 1 | 152* | 536 | OncoSeek Amplicon Panel |
|  |  |  |  | I14_0002 | 203 + 409 | carcinoma | 0.28 | 1.44 | 152* | 372 | OncoSeek Amplicon Panel |
| s4 | 30 | 10 | ER^+^ HER2^−^ breast from surgery | I12_0019 | 110 | stromal | 0.74 | 1 | 17 | 20 | LibPrep + LowPass WGS |
|  |  |  |  | I12_0020 | 110 | carcinoma | 0.74 | NA | 20 | 20 | LibPrep + LowPass WGS |
|  |  |  |  | I14_0003 | 110 | stromal | 0.74 | 1 | 41* | 51 | OncoSeek Amplicon Panel |
|  |  |  |  | I14_0004 | 110 | carcinoma | 0.74 | NA | 48 | 51 | OncoSeek Amplicon Panel |
| s5 | 95 | 6 | ER^+^ PR^+^ HER2^−^ breast from surgery | I14_0005 | 430 + 115 | stromal | 0.37 | 1 | 371 | 162 | OncoSeek Amplicon Panel |
|  |  |  |  | I14_0006 | 500 + 81 | carcinoma | 0.37 | 1.53 | 231 | 106 | OncoSeek Amplicon Panel |
| s6 | 80 | 7 | ER^+^ PR^+^ HER2^+^ breast from surgery | I12_0010 | 231 + 192 | stromal | 0.35 | 1 | 231 | 43 | LibPrep + LowPass WGS |
|  |  |  |  | I12_0011 | 78 + 31 | carcinoma | 0.35 | 0.505 | 78 | 85 | LibPrep  + LowPass WGS |
|  |  |  |  | I12_0012 | 117 + 18 | carcinoma | 0.35 | 1.05 | 117 | 41 | LibPrep + LowPass WGS |
|  |  |  |  | I12_0013 | 12 + 22 | carcinoma | 0.35 | 1.8 | 12* | 24 | LibPrep + LowPass WGS |
|  |  |  |  | I14_0007 | 440 + 231 | stromal | 0.35 | 1 | 432 | 171 | OncoSeek Amplicon Panel |
|  |  |  |  | I14_0008 | 230 + 59 | carcinoma | 0.35 | 0.96 | 266 | 179 | OncoSeek Amplicon Panel |
| s7 | 50 | 3 | ER^+^ PR^+^ HER^−^ breast biopsy | I14_0009 | 141 + 71 | stromal | 0.33 | 1 | 156* | 182 | OncoSeek Amplicon Panel |
|  |  |  |  | I14_0010 | 400 + 579 | carcinoma | 0.33 | 1 | 356 | 196 | OncoSeek Amplicon Panel |
| s8 | 80 | 3 | High-Grade Serous Ovarian | I12_0021 | 131 + 283 | stromal | 0.62 | 1 | 35 | 24 | LibPrep + LowPass WGS |
|  |  |  |  | I12_0022 | 116 + 339 | carcinoma | 0.62 | 1.51 | 25 | 16 | LibPrep + LowPass WGS |
|  |  |  |  | I14_0012 | 131 + 283 | stromal | 0.62 | 1 | 88 | 97 | OncoSeek Amplicon Panel |
|  |  |  |  | I14_0013 | 116 + 339 | carcinoma | 0.62 | 1.51 | 62 | 64 | OncoSeek Amplicon Panel |
| s9 | 70 | 10 | High-Grade Serous Ovarian | I12_0023 | 116 | stromal | 1.07 | 1 | 24 | 9 | LibPrep + LowPass WGS |
|  |  |  |  | I12_0024 | 150 + 285 | carcinoma | 1.07 | 1.64 | 25 | 5 | LibPrep + LowPass WGS |
|  |  |  |  | I14_0014 | 116 | stromal | 1.07 | 1 | 95 | 35 | OncoSeek Amplicon Panel |
|  |  |  |  | I14_0015 | 150 + 285 | carcinoma | 1.07 | 1.64 | 69 | 21 | OncoSeek Amplicon Panel |
| s10 |  | 8 | High-Grade Serous Ovarian | I12_0025 | 500 + 178 | stromal | 0.06 | 1 | 413* | 625 | LibPrep + LowPass WGS |
|  |  |  |  | I12_0026 | 322 + 18 | carcinoma | 0.06 | 1.51 | 290* | 414 | LibPrep + LowPass WGS |
| s11 | 10 | 7 | ER^+^ HER2^−^ breast from surgery | L68_003 | 70 + 505 | stromal | 0.36 | 1 | 53 | 42 | LibPrep + LowPass WGS |
|  |  |  |  | L68_004 | 46 + 8 | carcinoma | 0.36 | 0.959 | 46 | 43 | LibPrep + LowPass WGS |
| s12 | 15 | 7 | Lymph node from axillary node | L68_005 | 70 + 539 | stromal | 0.31 | 1 | 60 | 48 | LibPrep + LowPass WGS |
|  |  |  |  | L68_006 | 44 + 11 | carcinoma | 0.31 | 1.01 | 47 | 48 | LibPrep + LowPass WGS |

Tissue type, tumour cellularity and age of each tissue; number of cells for each population after the first selection (overall score > 54), and secondary selection (overall score < 54) in the DEPArray report, cell type, the DEPArray FFPE QC score, DNA Index (DI) calculated by DEPArray, the number of cells we actually pooled (usually entirely from the first selection but in some cases we used additional cells from the second selection), the theoretical number of cells required for meaningful genomic analysis and the downstream application are shown. The star symbol (*) highlights samples with libraries of fewer cells compared to the required number.

**Table S2.** Effective amplifiable template (EAT) minimum for each downstream application, depending on different QC scores.

| **Protocol** | **EAT**  **(min)** | **Diploid cell count QC ≤ 0.2** | **Diploid cell count QC ≤ 0.5** | **Diploid cell count QC ≤ 0.8** |
| --- | --- | --- | --- | --- |
| **DEPArray LibPrep + whole exome/genome sequencing** | 300 | 750 | 300 | 188 |
| **DEPArray OncoSeek amplicon panel** | 120 | 300 | 120 | 75 |
| **DEPArray LibPrep + LowPass WGS** | 30 | 75 | 30 | 19 |
| **Ampli1** **WGA + Ampli1 LowPass** | 1 | na | ~1 | 1 |

EAT, Effectively Amplifiable Template; QC, quality control; WGS, whole-genome sequencing; WGA, whole-genome amplification; na, not applicable.

**Table S3**. Comparison of somatic copy number alterations (sCNA) called by LowPass whole-genome sequencing (WGS) and the OncoSeek panel in the four samples run with both technologies.

| **Sample 3** | Low Pass | Oncomine |  | **Sample 8** | Low Pass | Oncomine |
| --- | --- | --- | --- | --- | --- | --- |
|  | CN Fold Change | |  |  | CN Fold Change | |
| ***CCND1*** | 2 | 3.4* |  | ***ALK*** | 1.5 | 2.4* |
| ***ERBB2*** | 2 | 1.6 |  | ***MYC*** | 4 | 2* |
| ***FGFR1*** | 3 | 4.3* |  |  | | |
|  | | |  |  |  |  |
| **Sample 6** | Low Pass | Oncomine |  | **Sample 9** | Low Pass | Oncomine |
|  | CN Fold Change | |  |  | CN Fold Change | |
| ***BRAF*** | 1.5 | 2.3* |  | ***CCND1*** | 1.5 | 1.9 |
| ***EGFR*** | 0.5 | 0.4 |  | ***ERBB2*** | 2.5 | 6.1* |
| ***ERBB2*** | 3 | 30.6* |  | ***FGFR1*** | 0.5 | 0.5 |
| ***FGFR2*** | 1.5 | 1.8 |  | ***FGFR2*** | 0.5 | 0.5 |
| ***MYC*** | 2.5 | 1.5 |  | ***MYC*** | 4 | 1.9 |
| ***PDGFRA*** | 0.5 | 0.1 |  | ***PIK3CA*** | 3.5 | 1.5 |

The 11 overlapping genes included in the panel and also present in the 93 BC-related protein-coding genes with probable driver mutations were: *BRAF, CCND1, CDK6, EGFR, ERBB2, FGFR1, FGFR2, KRAS, MYC, PDGFRA, PIK3CA*. Fold change for all somatic copy number alterations (sCNA) (only amplifications can be called, i.e. CN Fold change ≥ 2) identified by the OncoSeek panel (highlighted with star), and all gains, amplifications and losses detected by low-pass WGS are shown. For OncoSeek, sCNA fold changes > 1.5 for gains/amplifications and < 0.5 for losses between stromal and tumour populations are included.
